# HLA-DRB1 and HLA-DQB1 genetic polymorphisms and susceptibility to coronary atherosclerosis in a Northeast Chinese Population: A case-control study

**DOI:** 10.1371/journal.pone.0353906

**Published:** 2026-07-23

**Authors:** Miaomiao Niu, Jing Gao, Xiaodong Han, Ying Dong, Hui Wang, Yue Zhang, Dongming Zhao, Zijian Wang, Fang Qi, Feng Wang, Qiuye Meng, Jinjiao Geng, Shuqi Wu, Ying Wang, Ying Zhang, Chaoyang Guo, Hua Chen

**Affiliations:** 1 Medical Innovation Research Department, Chinese PLA General Hospital, Beijing, China; 2 Department of Cardiology, Liaoyuan municipal central hospital of Jilin Province, Jilin, China; UNAM FACMED: Universidad Nacional Autonoma de Mexico Facultad de Medicina, MEXICO

## Abstract

Atherosclerosis is a chronic inflammatory disease with increasing prevalence in Northeast China, where HLA class II molecules play an important immunoregulatory role. However, the contribution of specific HLA-DRB1 and HLA-DQB1 alleles to AS susceptibility in this population remains incompletely characterized, necessitating novel biomarkers for early detection. In this case-control study of 209 participants, HLA-DRB1 and HLA-DQB1 allele and phenotypic haplotype distributions were compared using odds ratios with 95% confidence intervals and Bonferroni correction for multiple comparisons. A total of 28 HLA-DRB1 and 12 HLA-DQB1 alleles were analyzed. The lowest *P* value for HLA-DRB1 was observed for DRB1*07:01 (*P* = 0.077, corrected *P* = 1.000; OR = 1.994, 95% CI: 0.955–4.164), and that for HLA-DQB1 was observed for DQB1*02:02 (*P* = 0.150, corrected *P* = 1.000; OR = 1.739, 95% CI: 0.850–3.558). Neither reached statistical significance, though both trended upward in the AS-susceptible group (DRB1*07:01: 12.59% vs. 6.76%; DQB1*02:02: 12.22% vs. 7.43%). The DRB1*07:01-DQB1*02:02 phenotypic haplotype was more frequent in the AS-susceptible group than in the control group (22.22% vs. 10.81%), with an OR of 2.357 (95% CI: 1.019–5.452). Although the association did not survive Bonferroni correction (corrected *P* = 1.000), the effect size suggested the signal was unlikely to be a trivial statistical artifact. In conclusion, the DRB1*07:01-DQB1*02:02 phenotypic haplotype was identified as a candidate risk marker for AS in the Northeast Chinese population, warranting validation in larger cohorts.

## Introduction

Atherosclerosis (AS) is a vascular disorder marked by the accumulation of lipids, persistent inflammation, and the development of fibrous plaques within the walls of arteries. It is a predominant contributor to cardiovascular diseases and poses a significant threat to global public health [1]. In recent years, shifts in lifestyle choices and environmental influences have led to a rising incidence of coronary atherosclerosis, particularly in regions like Northeast China [2]. This trend has intensified the strain on local healthcare systems and social security, underscoring the urgent necessity for enhanced preventive and therapeutic strategies. Despite the progress made in treatment options, including lipid-lowering agents, antihypertensive drugs, and antiplatelet therapies, considerable obstacles persist regarding early diagnosis and tailored management, particularly among high-risk individuals for whom traditional approaches may fall short.

Growing evidence has connected human leukocyte antigen (HLA) genes to the development of atherosclerosis, highlighting their potential involvement in immunogenetic pathways [3–7]. Earlier studies have identified associations between particular HLA alleles, especially those located within the HLA-DR and -DQ genes, and a variety of autoimmune and cardiovascular conditions, indicating a possible association with the advancement of atherosclerosis [8,9]. Nevertheless, the exact relationship between the HLA-DR and HLA-DQ subregions and coronary atherosclerosis remains inadequately elucidated, warranting further investigation.

Consequently, this study aims to systematically evaluate the influence of prevalent HLA-DR and HLA-DQ genotypes or phenotypic haplotypes on the incidence of atherosclerosis within the Northeast Chinese population through a case-control study methodology. We anticipate that the findings of this investigation will establish a scientific foundation for early detection and personalized prevention strategies for atherosclerosis, while also providing novel insights and avenues for future research in related domains.

## Materials and methods

### Study participants

This study enrolled 209 participants aged 30−60 years (mean age: 40; 136 males and 73 females, Table 1) from the Department of Cardiology, Liaoyuan Municipal Central Hospital of Jilin Province, China, between October 2023 and August 2024. Geographically, Jilin is part of Northeast China, a region comprising the provinces of Liaoning, Heilongjiang, and Jilin itself. The AS-susceptible group consisted of 135 patients with coronary atherosclerosis, defined as more than 50% stenosis in at least one major coronary artery, including the left anterior descending, right coronary, left circumflex, or left main, as confirmed by coronary computed tomography angiography (CTA). Among these patients, 64 (47.4%) had single-vessel disease, 51 (37.8%) had double-vessel disease, and 20 (14.8%) had triple-vessel disease. The control group comprised 74 non-susceptible individuals with less than 20% stenosis in all major coronary arteries, verified by the same imaging modality. Baseline clinical characteristics of both groups are presented in Table 1. The study protocol was approved by the Medical Research Ethics Committee of Liaoyuan Municipal Central Hospital of Jilin Province (Approval No: L2023-08–001). All participants provided written informed consent and completed a standardized epidemiological survey.

**Table 1:** Comparison of clinical characteristics between the AS-susceptible group and the control group (n = 209).

Variables	AS-susceptible group (n = 135)		Control group (n = 74)		*P value*
	N	%	N	%	–
Male	105	77.5	31	37.1	< 0.001^#^
Female	30	22.5	43	62.9	< 0.001^#^
Age range	30-60	–	30-60	–	–
Age (mean±SD)	52.1 ± 6.8	–	51.0 ± 7.3	–	0.265
**Clinical and laboratory data**					
Hypertension	50	37.0	7	9.5	< 0.001^#^
Diabetes	41	30.4	5	6.8	< 0.001^#^
Hypertriglyceridemia	67	49.6	32	43.2	0.377
Hypercholesterolemia	15	11.1	11	14.9	0.432
Hyper-LDL cholesterolemia	21	15.6	16	21.6	0.272
Hyper-HDL cholesterolemia	0	0.0	1	1.4	0.354
Hypo-HDL cholesterolemia	75	55.6	26	35.1	0.005^#^
Triglyceride (mmol/L) (mean±SD)	2.1 ± 1.7	–	1.9 ± 1.6	–	0.038^#^
Cholesterol (mmol/L) (mean±SD)	4.2 ± 1.5	–	4.6 ± 1.0	–	< 0.001^#^
High density lipoprotein (mmol/L) (mean±SD)	1.0 ± 0.3	–	1.2 ± 0.3	–	< 0.001^#^
Low density lipoprotein (mmol/L) (mean±SD)	2.5 ± 1.3	–	2.8 ± 0.8	–	0.065
**Smoking status**					
Current	19	14.1	15	20.3	0.279
Ex-smoker	2	1.5	0	0.0	0.508
Never-smoker	19	14.1	47	63.5	< 0.001^#^-
Unknown	95	70.4	12	16.2	< 0.001^#^
**Angiographic characteristics**					
None	0	0.0	59	79.7	< 0.001^#^
Single vessel disease	64	47.4	15	20.3	0.0004^#^
Two vessel disease	51	37.8	0	0.0	< 0.001^#^
Three vessel disease	20	14.8	0	0.0	< 0.001^#^

# represents *P* value no more than 0.05.

### Methods

#### Sample collection and processing.

2 mL fasting peripheral venous blood was collected from each participant in the morning using vacuum EDTA anticoagulant tubes. After gentle mixing, samples were aliquoted into 500 μL tubes and stored at −80 °C for further analysis.

#### HLA-DRB1 and HLA-DQB1 genotyping and phenotypic haplotype assignment.

Genomic DNA was extracted from whole blood using the Blood Genomic DNA Extraction Kit (Tiangen Biotech Co., Ltd, China) according to the manufacturer’s instructions. DNA samples were sent to Beijing Bofurui Medical Laboratory Co., Ltd for HLA-DRB1 and HLA-DQB1 genotyping using next-generation sequencing (NGS). In the absence of family data or molecular phasing, the chromosomal phase of DRB1 and DQB1 allele pairs could not be definitively established. Accordingly, we employ the term “phenotypic haplotype” throughout this study to refer to the co-occurrence of a specific DRB1 allele and a specific DQB1 allele at the individual level, recognizing that these combinations do not necessarily represent true haplotypes in phase. The frequency of each phenotypic haplotype was calculated separately for each group as the number of individuals carrying both the DRB1 and DQB1 alleles of interest, divided by the total number of individuals in each group (n = 135 for the AS-susceptible group; n = 74 for the control group). These phenotypic haplotypes were used for all subsequent association analyses.

#### Clinical data collection and measurements.

Basic information including gender, age, blood pressure, and medical history was collected by clinicians. Laboratory parameters such as blood glucose, triglyceride (TG), total cholesterol (TC), low-density lipoprotein cholesterol (LDL-C), and high-density lipoprotein cholesterol (HDL-C) were measured by the Clinical Laboratory Department of Liaoyuan Municipal Central Hospital. All data were recorded by clinicians. Coronary CT angiography (CTA) was performed by the hospital’s Imaging Department. The number of stenotic coronary vessels and the degree of stenosis were diagnosed by clinicians based on imaging results.

### Statistical analysis

Statistical analyses were performed using SPSS software (version 17.0, SPSS Inc., Chicago, IL, USA). Continuous variables with a normal distribution, including age and lipid profiles, were expressed as mean ± standard deviation and compared between groups using the independent samples t-test. Hardy-Weinberg equilibrium (HWE) using the “Basic statistics” tool, available on the HLA-net website (https://hla-net.eu/tools/basic-statistics/), was assessed in the control group to evaluate genotyping quality and population stratification. Categorical data, including the frequencies of HLA-DRB1 and HLA-DQB1 alleles and phenotypic haplotype distributions, were compared using the χ² test or Fisher’s exact test, as appropriate.

To account for multiple comparisons, raw *P* values were corrected using the Bonferroni method by multiplying by the number of phenotypic haplotypes tested. Corrected *P* values < 0.05 were considered statistically significant.

The strength of association between each allele or phenotypic haplotype and disease susceptibility were quantified by calculating odds ratios (ORs) with 95% confidence intervals (CIs). An OR > 1 with a 95% CI excluding 1 was considered a risk factor, whereas an OR < 1 with a 95% CI excluding 1 was considered a protective factor.

Within the AS-susceptible group, the χ² test or Fisher’s exact test was further used to evaluate associations between high-frequency alleles or phenotypic haplotypes and clinical parameters, including hypertension, diabetes and hyperlipidemia, and the number of affected coronary vessels. *P* value no more than 0.05 was considered statistically significant.

## Results

### Comparison of Clinical characteristics between AS-susceptible and non-susceptible groups

As summarized in Table 1, comparative analysis between the two groups revealed a significantly higher proportion of males in the AS-susceptible group compared to the control group (77.5% vs. 37.1%), which is consistent with the established gender disparity in the pathogenesis of coronary atherosclerosis. No significant difference was observed in age distribution between the groups.

Regarding comorbidities, the prevalences of both hypertension and diabetes were significantly higher in the AS-susceptible group than in the control group (37.0% vs. 9.5% and 30.4% vs. 6.8%, respectively), aligning with the well-recognized epidemiological association of these conditions with increased AS risk.

In terms of lipid profiles, the AS-susceptible group exhibited a higher mean TG level compared to controls (2.1 ± 1.7 vs. 1.9 ± 1.6), although the difference in the incidence of hypertriglyceridemia was not statistically significant. Notably, the mean TC and HDL-C levels were significantly lower in the AS-susceptible group (4.2 ± 1.5 vs. 4.6 ± 1.0 and 1.0 ± 0.3 vs. 1.2 ± 0.3, respectively). The mean LDL-C level was also slightly lower (2.5 ± 1.3 vs. 2.8 ± 0.8), though without statistical significance. These observations may be may be partly explained by the higher proportion of patients in the AS-susceptible group receiving lipid-lowering therapy, although detailed medication data were not available for all participants. With respect to the incidence of dyslipidemias, the rate of hypo-HDL was higher in the AS-susceptible group (55.6% vs. 35.1%). In contrast, no statistically significant differences were found between the two groups in the incidence of hypertriglyceridemia, hypercholesterolemia, hyper-LDL, or hyper-HDL.

### Analysis of HLA-DRB1 and HLA-DQB1 allele distributions

The distributions of HLA-DRB1 and HLA-DQB1 alleles were compared between the AS-susceptible group (n = 135, 270 alleles) and the healthy control group (n = 74, 148 alleles). A total of 28 HLA-DRB1 and 12 HLA-DQB1 alleles were analyzed. Hardy-Weinberg equilibrium testing in the control group showed no deviation from HWE (*P* = 1.0000, S1 File). These results indicate that the genotypic distributions were consistent with Mendelian expectations, supporting the reliability of the subsequent allele distribution comparisons. As shown in Table 2, the most frequent HLA-DRB1 alleles in the AS-susceptible group were DRB1*07:01 (12.59%), DRB1*09:01 (6.30%), and DRB1*04:05 (4.07%). In the control group, the most common alleles were DRB1*09:01 (9.46%), DRB1*07:01 (6.76%), and DRB1*03:01 (4.05%). For the HLA-DQB1 locus, the most common alleles in the AS-susceptible group were DQB1*03:01 (16.67%), DQB1*02:02 (12.22%), and DQB1*03:03 (6.30%). In the control group, the predominant alleles were DQB1*03:01 (17.57%), DQB1*02:02 (7.43%), DQB1*03:03 (6.08%), and DQB1*03:02 (6.08%).

**Table 2:** The distributions of HLA-DRB1 and HLA-DQB1 alleles in the AS-susceptible group and the control group and their association with AS susceptibility.

HLA Alleles	AS-susceptible group (n = 135 individuals)		Control group (n = 74 individuals)		Raw *P value*	Corrected *P* value	OR	95% CI
	Allelic count (2n = 270)	Frequency (%)	Allelic count (2n = 148)	Frequency (%)				
HLA-DRB1								
DRB1*01:01	3	1.11	2	1.35	1.000	1.000	0.821	0.136–4.956
DRB1*01:02	1	0.37	0	0.00	1.000	1.000	1.642	0.066–40.763
DRB1*03:01	5	1.85	6	4.05	0.196	1.000	0.447	0.134–1.487
DRB1*04:01	5	1.85	4	2.70	0.735	1.000	0.681	0.181–2.557
DRB1*04:02	0	0.00	2	1.35	0.149	1.000	0.109	0.006–2.049
DRB1*04:03	1	0.37	3	2.03	0.348	1.000	0.232	0.024–2.240
DRB1*04:04	8	2.96	1	0.68	0.152	1.000	4.488	0.558–36.119
DRB1*04:05	11	4.07	2	1.35	0.144	1.000	3.103	0.682–14.120
DRB1*04:06	8	2.96	4	2.70	1.000	1.000	1.100	0.325–3.720
DRB1*04:08	1	0.37	0	0.00	1.000	1.000	1.642	0.066–40.763
DRB1*07:01	34	12.59	10	6.76	0.077	1.000	1.994	0.955–4.164
DRB1*08:01	0	0.00	1	0.68	0.354	1.000	0.183	0.007–4.602
DRB1*08:02	1	0.37	1	0.68	1.000	1.000	0.548	0.034–8.868
DRB1*08:03	10	3.70	4	2.70	0.768	1.000	1.383	0.426–4.488
DRB1*09:01	17	6.30	14	9.46	0.266	1.000	0.646	0.307–1.357
DRB1*10:01	2	0.74	3	2.03	0.374	1.000	0.364	0.060–2.202
DRB1*11:01	7	2.59	3	2.03	0.741	1.000	1.283	0.327–5.031
DRB1*11:04	1	0.37	0	0.00	1.000	1.000	1.642	0.066–40.763
DRB1*12:01:01G	4	1.48	4	2.70	0.506	1.000	0.547	0.135–2.214
DRB1*12:02	6	2.22	2	1.35	0.723	1.000	1.662	0.332–8.329
DRB1*13:01	1	0.37	1	0.68	1.000	1.000	0.548	0.034–8.868
DRB1*13:02	3	1.11	3	2.03	0.671	1.000	0.548	0.109–2.759
DRB1*13:12	0	0.00	1	0.68	0.354	1.000	0.183	0.007–4.602
DRB1*14:04	0	0.00	1	0.68	0.354	1.000	0.183	0.007–4.602
DRB1*14:05	1	0.37	0	0.00	1.000	1.000	1.642	0.066–40.763
DRB1*14:07	1	0.37	0	0.00	1.000	1.000	1.642	0.066–40.763
DRB1*14:54	1	0.37	0	0.00	1.000	1.000	1.642	0.066–40.763
DRB1*15:01	3	1.11	2	1.35	1.000	1.000	0.821	0.136–4.956
HLA-DQB1								
DQB1*02:01	5	1.85	6	4.05	0.196	1.000	0.447	0.134–1.487
DQB1*02:02	33	12.22	11	7.43	0.150	1.000	1.739	0.850–3.558
DQB1*03:01	45	16.67	26	17.57	0.827	1.000	0.940	0.550–1.606
DQB1*03:02	11	4.07	9	6.08	0.466	1.000	0.659	0.266–1.632
DQB1*03:03	17	6.30	9	6.08	1.000	1.000	1.037	0.448–2.401
DQB1*03:22	1	0.37	0	0.00	1.000	1.000	1.642	0.066–40.763
DQB1*04:01	7	2.59	2	1.35	0.507	1.000	1.946	0.400–9.461
DQB1*05:01	2	0.74	3	2.03	0.374	1.000	0.364	0.060–2.202
DQB1*05:02	4	1.48	3	2.03	0.717	1.000	0.731	0.161–3.316
DQB1*05:03	3	1.11	1	0.68	1.000	1.000	1.653	0.172–15.916
DQB1*06:01	5	1.85	2	1.35	0.732	1.000	1.383	0.265–7.217
DQB1*06:02	2	0.74	2	1.35	0.625	1.000	0.548	0.076–3.928

Allelic counts are based on 2n. Chi-square test or Fisher’s exact test (when expected cell count < 5) was used for comparison between groups. OR values and 95% CIs were calculated using Haldane correction (adding 0.5 to each cell of the 2 × 2 contingency table) to address zero counts. A two-sided *P* < 0.05 was considered statistically significant. Raw *P* values were corrected using the Bonferroni method, and corrected *P* values > 1.000 were truncated to 1.000.

In the allele distribution comparison, a consistent upward trend was observed for two HLA alleles in the AS-susceptible group relative to the control group. The carrier frequency of DRB1*07:01 increased from 6.76% in the control group to 12.59% in the AS-susceptible group, while that of DQB1*02:02 rose from 7.43% to 12.22%. Despite this directional consistency, neither allele reached statistical significance at the nominal level. The lowest *P* value among all DRB1 alleles was observed for DRB1*07:01 (*P* = 0.077, OR = 1.994, 95% CI: 0.955–4.164), with an odds ratio approaching 2.0 and a lower confidence bound only marginally below unity. Among DQB1 alleles, DQB1*02:02 yielded the smallest *P* value (*P* = 0.150, OR = 1.739, 95% CI: 0.850–3.558). To adjust for multiple comparisons across 40 independent allele tests, a Bonferroni correction was applied, after which all *P* values were adjusted to 1.000, indicating that no single allele retained a statistically significant association with AS susceptibility.

### Analysis of HLA-DRB1-DQB1 phenotypic haplotype frequencies

Because family data or molecular phasing were not available, the chromosomal assignment of DRB1 and DQB1 allele pairs could not be determined. Therefore, throughout this study, we use the term “phenotypic haplotype” to denote the co-occurrence of a DRB1 allele and a DQB1 allele at the individual level, acknowledging that these do not necessarily represent true haplotypes. A total of 56 HLA-DRB1-DQB1 phenotypic haplotypes were compared between AS-susceptible group and control group in Table 3. After Bonferroni correction for multiple comparisons (corrected *P* = raw *P* × 56), no phenotypic haplotype showed a statistically significant association with AS susceptibility. The most frequently observed phenotypic haplotype in the AS group was DRB1*07:01-DQB1*02:02, which was present in 30/135 (22.22%) AS-susceptible group and 8/74 (10.81%) control group. This phenotypic haplotype exhibited a nominal association with AS before correction (raw *P* = 0.041, OR = 2.357, 95% CI: 1.019–5.452). However, the significance did not survive Bonferroni correction (corrected *P* = 1.000). The phenotypic haplotype DRB1*04:03-DQB1*03:02 was detected exclusively in the control group (3/74, 4.05%) and was absent in the AS-susceptible group (0/135). The difference was also not significant after correction (raw *P* = 0.047, corrected *P* = 1.000; OR = 0.075, 95% CI: 0.004–1.471). For all other phenotypic haplotypes, no significant differences were observed between the two groups.

**Table 3:** The carrier frequencies of HLA-DRB1-DQB1 pairs in the AS-susceptible group and the control group and their association with AS susceptibility.

HLA-DRB1-DQB1 phenotypic haplotype	AS-susceptible group (n = 135 individuals)		Control group (n = 74 individuals)		Raw *P value*	*Corrected P value*	OR	95% CI
	N	Frequency (%)	N	Frequency (%)				
DRB1*07:01-DQB1*02:02	30	22.22	8	10.81	0.041#	1.000	2.357	1.019-5.452
DRB1*09:01-DQB1*03:03	10	7.41	7	9.46	0.604	1.000	0.769	0.280-2.111
DRB1*09:01-DQB1*03:01	7	5.19	7	9.46	0.267	1.000	0.524	0.177-1.555
DRB1*04:06-DQB1*03:02	7	5.19	4	5.41	0.758	1.000	0.958	0.270-3.401
DRB1*11:01-DQB1*03:01	7	5.19	3	4.05	0.746	1.000	1.296	0.324-5.186
DRB1*04:05-DQB1*04:01	7	5.19	2	2.70	0.492	1.000	1.979	0.399-9.817
DRB1*12:02-DQB1*03:01	6	4.44	2	2.70	0.713	1.000	1.690	0.331-8.632
DRB1*08:03-DQB1*03:01	6	4.44	1	1.35	0.419	1.000	3.441	0.407-29.106
DRB1*03:01-DQB1*02:01	5	3.70	6	8.11	0.207	1.000	0.435	0.128-1.478
DRB1*04:01-DQB1*03:01	4	2.96	2	2.70	1.000	1.000	1.102	0.196-6.186
DRB1*04:04-DQB1*03:01	4	2.96	1	1.35	0.649	1.000	2.238	0.245-20.418
DRB1*07:01-DQB1*03:03	4	2.96	1	1.35	0.649	1.000	2.238	0.245-20.418
DRB1*01:01-DQB1*03:01	3	2.22	0	0.00	0.549	1.000	4.908	0.250-96.345
DRB1*04:04-DQB1*03:02	3	2.22	0	0.00	0.549	1.000	4.908	0.250-96.345
DRB1*12:01:01G-DQB1*03:01	2	1.48	4	5.41	0.196	1.000	0.263	0.047-1.474
DRB1*08:03-DQB1*06:01	2	1.48	2	2.70	0.594	1.000	0.544	0.075-3.964
DRB1*10:01-DQB1*05:01	2	1.48	2	2.70	0.594	1.000	0.544	0.075-3.964
DRB1*13:02-DQB1*06:01	2	1.48	0	0.00	1.000	1.000	3.593	0.170-75.946
DRB1*04:05-DQB1*03:03	2	1.48	0	0.00	1.000	1.000	3.593	0.170-75.946
DRB1*04:01-DQB1*02:02	1	0.74	1	1.35	0.536	1.000	0.546	0.034-8.884
DRB1*08:03-DQB1*05:02	1	0.74	1	1.35	0.536	1.000	0.546	0.034-8.884
DRB1*13:02-DQB1*05:02	1	0.74	1	1.35	0.536	1.000	0.546	0.034-8.884
DRB1*15:01-DQB1*05:02	1	0.74	1	1.35	0.536	1.000	0.546	0.034-8.884
DRB1*15:01-DQB1*06:02	1	0.74	1	1.35	0.536	1.000	0.546	0.034-8.884
DRB1*01:02-DQB1*03:01	1	0.74	0	0.00	1.000	1.000	2.017	0.081-50.043
DRB1*04:03-DQB1*03:01	1	0.74	0	0.00	1.000	1.000	2.017	0.081-50.043
DRB1*04:04-DQB1*02:02	1	0.74	0	0.00	1.000	1.000	2.017	0.081-50.043
DRB1*04:05-DQB1*02:02	1	0.74	0	0.00	1.000	1.000	2.017	0.081-50.043
DRB1*04:05-DQB1*03:01	1	0.74	0	0.00	1.000	1.000	2.017	0.081-50.043
DRB1*04:06-DQB1*03:01	1	0.74	0	0.00	1.000	1.000	2.017	0.081-50.043
DRB1*04:08-DQB1*03:01	1	0.74	0	0.00	1.000	1.000	2.017	0.081-50.043
DRB1*08:02-DQB1*03:02	1	0.74	0	0.00	1.000	1.000	2.017	0.081-50.043
DRB1*08:03-DQB1*03:03	1	0.74	0	0.00	1.000	1.000	2.017	0.081-50.043
DRB1*11:04-DQB1*03:01	1	0.74	0	0.00	1.000	1.000	2.017	0.081-50.043
DRB1*12:01:01G-DQB1*03:22	1	0.74	0	0.00	1.000	1.000	2.017	0.081-50.043
DRB1*12:01:01G-DQB1*05:03	1	0.74	0	0.00	1.000	1.000	2.017	0.081-50.043
DRB1*13:01-DQB1*06:02	1	0.74	0	0.00	1.000	1.000	2.017	0.081-50.043
DRB1*14:05-DQB1*05:03	1	0.74	0	0.00	1.000	1.000	2.017	0.081-50.043
DRB1*14:07-DQB1*05:02	1	0.74	0	0.00	1.000	1.000	2.017	0.081-50.043
DRB1*14:54-DQB1*05:03	1	0.74	0	0.00	1.000	1.000	2.017	0.081-50.043
DRB1*15:01-DQB1*06:01	1	0.74	0	0.00	1.000	1.000	2.017	0.081-50.043
DRB1*04:03-DQB1*03:02	0	0.00	3	4.05	0.047#	1.000	0.075	0.004-1.471
DRB1*01:01-DQB1*02:02	0	0.00	1	1.35	0.354	1.000	0.181	0.007-4.489
DRB1*01:01-DQB1*03:03	0	0.00	1	1.35	0.354	1.000	0.181	0.007-4.489
DRB1*04:01-DQB1*03:02	0	0.00	1	1.35	0.354	1.000	0.181	0.007-4.489
DRB1*04:02-DQB1*02:02	0	0.00	1	1.35	0.354	1.000	0.181	0.007-4.489
DRB1*04:02-DQB1*03:02	0	0.00	1	1.35	0.354	1.000	0.181	0.007-4.489
DRB1*07:01-DQB1*03:01	0	0.00	1	1.35	0.354	1.000	0.181	0.007-4.489
DRB1*08:01-DQB1*03:01	0	0.00	1	1.35	0.354	1.000	0.181	0.007-4.489
DRB1*08:02-DQB1*03:01	0	0.00	1	1.35	0.354	1.000	0.181	0.007-4.489
DRB1*10:01-DQB1*03:01	0	0.00	1	1.35	0.354	1.000	0.181	0.007-4.489
DRB1*13:02-DQB1*05:01	0	0.00	1	1.35	0.354	1.000	0.181	0.007-4.489
DRB1*13:01-DQB1*03:01	0	0.00	1	1.35	0.354	1.000	0.181	0.007-4.489
DRB1*13:12-DQB1*03:01	0	0.00	1	1.35	0.354	1.000	0.181	0.007-4.489
DRB1*13:02-DQB1*06:02	0	0.00	1	1.35	0.354	1.000	0.181	0.007-4.489
DRB1*14:04-DQB1*05:03	0	0.00	1	1.35	0.354	1.000	0.181	0.007-4.489

Phenotypic haplotypes were defined as the co-occurrence of a DRB1 allele and a DQB1 allele at the individual level, regardless of chromosomal phase. N represented as the number of individuals carrying the pair. Frequencies were calculated as the number of individuals carrying the specific allele pair divided by the total number of individuals in each group (AS: n = 135; control: n = 74). The chi-square test or Fisher’s exact test (when expected cell count < 5) was used for comparison between groups. OR and 95% CI were calculated using the Woolf method with Haldane’s correction for zero cells. A two-sided *P* < 0.05 was considered statistically significant. Raw *P* values were corrected using the Bonferroni method (corrected *P* = raw *P* × number of haplotypes tested), and corrected *P* values > 1.000 were truncated to 1.000. # indicated nominal significance (*P* < 0.05) before correction, which did not remain significant after Bonferroni correction.

### Correlation analysis between differential alleles/phenotypic haplotypes and clinical indicators

Further analysis was conducted among the 135 AS-susceptible patients to assess the correlations between the DRB1*07:01 allele, DQB1*02:02 allele, and DRB1*07:01-DQB1*02:02 phenotypic haplotype and clinical features including hypertension, hypertriglyceridemia, high LDL-C, low HDL-C and the number of pathological coronary artery vessels. As shown in Table 4, no significant associations were observed between these genetic variants and any of the clinical characteristics. Correlation analysis between the DRB1*04:03-DQB1*03:02 phenotypic haplotype and clinical features was not performed, as this phenotypic haplotype was entirely absent in the AS-susceptible group. Furthermore, its nominal association with AS susceptibility was statistically fragile (raw *P* = 0.047, OR = 0.075, 95% CI: 0.004–1.471) and did not withstand multiple correction (corrected *P* = 1.000), rendering further stratified analyses unjustified.

**Table 4:** The relationship between DRB1 and DQB1 allele with clinical parameters and the number of affected coronary vessels within the AS-susceptible group.

HLA Alleles/Phenotypic haplotype	Hypertension			Diabetes			Hyper-TG			Hyper-TC			Hyper-LDL-C			Angiography status			
	Yes (n = 50)	No (n = 85)	*P* value	Yes (n = 41)	No (n = 94)	*P* value	Yes (n = 67)	No (n = 68)	*P* value	Yes (n = 15)	No (n = 120)	*P* value	Yes (n = 18)	No (n = 117)	*P* value	SVD (n = 64)	2VD (n = 51)	3VD (n = 20)	*P* value
DRB1*07:01 (n = 34)	12	22	0.808	11	23	0.771	13	21	0.124	4	30	1.000	5	29	0.785	13	16	5	0.398
DQB1*02:02 (n = 33)	11	22	0.612	10	23	0.992	15	18	0.581	5	28	0.396	4	29	0.814	14	15	4	0.570
DRB1*07:01-DQB1*02:02 (n = 30)	11	19	0.962	9	21	0.960	12	18	0.232	4	26	0.913	4	26	1.000	12	14	4	0.519

SVD = Single vessel disease, 2VD = Two vessel disease, 3VD = Three vessel disease. Chi-square test or Fisher’s exact test (Expected cells value less than 5) was used for comparison. ^#^ represents *P* value no more than 0.05.

## Discussion

Atherosclerosis is a chronic inflammatory disease characterized by endothelial dysfunction at susceptible arterial sites [1], which promotes subendothelial accumulation of apolipoprotein B (APOB)-rich low-density lipoprotein (LDL) particles [10–12]. Oxidized LDL triggers innate and adaptive immune activation, leading to leukocyte recruitment, fibrous tissue proliferation, and calcium deposition, ultimately driving plaque formation [12,13]. As a leading cause of cardiovascular diseases, atherosclerosis contributes substantially to global morbidity and mortality [14–16]. Recent lifestyle and dietary shifts have exacerbated its prevalence, particularly in Northeast China, posing significant public health and economic burdens [2,17–20]. A deeper understanding of its mechanisms and risk factors is essential for improving early detection and prevention [21,22].

Genetic factors, particularly within the human leukocyte antigen (HLA) region, are increasingly recognized for their role in immune regulation of atherosclerosis [3–5]. Genome-wide association studies (GWAS) have identified several HLA-linked variants associated with cardiovascular risk [23]. Clinical studies have also reported associations between specific HLA alleles and atherosclerosis. For example, Hamed et al. observed a higher frequency of HLA-A*02 in atherosclerosis patients [3], while Eder et al. linked HLA-B*13:02 and HLA-C*06:02 to more severe atherosclerosis in psoriatic individuals [24]. Hossein et al. suggested a potential protective effect of HLA-DRB1*01 in a cohort from southwestern Iran [4]. These findings collectively underscore the relevance of HLA polymorphisms in atherosclerosis, though the full spectrum of risk genotypes remains incompletely defined.

In the present study, we investigated HLA-DRB1 and DQB1 genotypes in a Northeast Chinese population, focusing on the DRB1*07:01-DQB1*02:02 phenotypic haplotype. The internal validity of our control group was robust. The carrier frequencies of DRB1*07:01 (6.76%) and DQB1*02:02 (7.43%) closely matched previously reported regional and national data (8.91–10.70% and 7.57%, respectively) [25,26]. Furthermore, Hardy-Weinberg equilibrium testing in the control group showed no deviation (*P* = 1.0000), confirming that genotypic distributions were consistent with Mendelian expectations. These observations effectively rule out population stratification or sampling bias as explanations for the case-control differences and provide a reliable baseline for subsequent analyses.

We observed the DRB1*07:01-DQB1*02:02 phenotypic haplotype at a frequency of 22.22% in the AS-susceptible group versus 10.81% in controls, corresponding to an odds ratio of 2.357 (95% CI: 1.019–5.452). Notably, the lower bound of the confidence interval exceeded 1.0, and the OR exceeded 2.0. This threshold is commonly considered indicative of potential clinical relevance in complex disease genetics, particularly for immunogenetic variants where moderate effects are common. Although the association did not survive Bonferroni correction for multiple comparisons, the effect size and confidence interval pattern suggest that the observed signal is unlikely to be a trivial statistical artifact. These findings warrant validation in larger, well-powered cohorts.

The observed association is biologically plausible. HLA class II molecules play a central role in activating CD4 ⁺ T cells and promoting pro-inflammatory cytokine production [27–29]. Supporting this, Takayuki et al. constructed a human DRB1*07:01 tetramer and identified ApoB peptide 2106 as the first regulatory T-cell epitope capable of inducing a specific T-cell response in human atherosclerosis [28]. This finding provideed a foundational basis for developing atherosclerosis vaccines [30]. Our results further emphasized the interplay between HLA class II gene polymorphisms and CD4 ⁺ T-cell activity in atherosclerosis pathogenesis.

We also examined whether the DRB1*07:01-DQB1*02:02 phenotypic haplotype was associated with metabolic parameters, including hypertension, dyslipidemia, and coronary lesion severity. No such associations were observed, suggesting that its risk effect operates independently of conventional metabolic pathways. HLA class II molecules primarily mediate exogenous antigen presentation and CD4 ⁺ T-cell activation [29]. Their pathogenic potential often requires environmental triggers, such as viral infections or dietary antigens, to become fully manifest [7,31,32]. The present study did not assess such gene-environment interactions, highlighting a direction for future integrated analyses.

The DRB1*04:03-DQB1*03:02 phenotypic haplotype was observed only in the control group (4.05%) and was absent in AS-susceptible group (raw *P* = 0.047, OR = 0.075, 95% CI: 0.004–1.471). However, the extremely low frequency renders this finding statistically unstable. The wide confidence interval and lack of significance after multiple-testing correction strongly suggest that this is a chance observation rather than a genuine protective association. Overall, our data do not support a significant role for any of the tested HLA-DRB1-DQB1 phenotypic haplotypes in AS susceptibility after rigorous correction.

Several additional limitations of this study should be acknowledged. First, all participants were recruited from a single center, which may limit the generalizability of our findings. Although our results primarily reflect the local population served by this institution, they offer valuable insights into the genetic architecture of the broader Northeast Chinese population, a region encompassing the provinces of Liaoning, Jilin, and Heilongjiang, which is characterized by a relatively homogeneous ethnic composition. Multicenter investigations incorporating additional recruitment sites across these three provinces are warranted to confirm whether the observed HLA-disease associations are consistent throughout the entire region. Second, in the absence of family data or molecular phasing, the chromosomal assignment of DRB1 and DQB1 allele pairs could not be definitively established. Consequently, throughout this study, we employ the term “phenotypic haplotype” to denote the co-occurrence of a DRB1 allele and a DQB1 allele at the individual level, while acknowledging that these do not necessarily represent true haplotypes in phase. Third, the lack of experimental validation precludes definitive conclusions regarding the underlying biological mechanisms. Functional studies, including in vitro assays or animal models, are required to establish causality and to delineate the molecular pathways through which specific HLA alleles might contribute to AS pathogenesis. Despite these limitations, our findings provide a foundation for future genetic and functional investigations in this understudied population.

## Conclusions

In conclusion, our findings identify the HLA-DRB1*07:01-DQB1*02:02 phenotypic haplotype as a candidate risk marker for AS in the Northeast Chinese population. Although the association did not survive correction for multiple testing, the consistent allele trends, population-validated controls, and effect size exceeding 2.0 collectively argue against dismissing the result as trivial. We advocate for validation in larger, independent cohorts with adequate power to detect modest genetic effects, alongside functional studies to elucidate the underlying immunopathological pathways.

## Supporting information

S1 File Hardy–Weinberg equilibrium (HWE) *P*-values for the control group. (PDF)

S1 Table Raw data for all statistical analyses and tables presented in the manuscript. (XLSX)
