# Supplementary material for: HLA-DRB1 and HLA-DQB1 genetic polymorphisms and susceptibility to coronary atherosclerosis in a Northeast Chinese Population: A case-control study
Source: PLoS One. 2026 Jul 23;21(7):e0353906. doi: 10.1371/journal.pone.0353906 (PMC13395462; doi:10.1371/journal.pone.0353906)
Supplement: S1 File — (PDF) [file pone.0353906.s001.pdf]

Table of p–values for
Hardy–Weinberg Equilibrium and
Global Linkage Disequilibrium

	Locus-0
Locus-0	1.0000

The diagonal contains the p–values for the Hardy–Weinberg equilibrium test, the upper triangular matrix those for the parametric global LD test and the lower triangular matrix those for the permutation non–parametric global LD test.
