# Supplementary material for: HLA-DRB1 and HLA-DQB1 genetic polymorphisms and susceptibility to coronary atherosclerosis in a Northeast Chinese Population: A case-control study
Source: PLoS One. 2026 Jul 23;21(7):e0353906. doi: 10.1371/journal.pone.0353906 (PMC13395462; doi:10.1371/journal.pone.0353906)
Supplement: Attachment — Submitted filename: Response to Reviewers.docx [file pone.0353906.s004.docx]

Dear Editor,

Thank you for giving us the opportunity to revise our manuscript (Manuscript ID: PONE-D-25-52254). We appreciate the time and effort that you and the reviewers have dedicated to providing valuable feedback on our work. We have carefully addressed all the comments raised by the editor and the reviewers, and the changes are highlighted in the revised manuscript. Below is our point-by-point response to each comment.

**Response to Journal Requirements：**

1. We have thoroughly reviewed our submission against the PLOS ONE style templates and guidelines. We confirm that the manuscript and all associated files now meet the journal’s formatting and file naming requirements.

2. Role of Funder Statement:

The National Natural Science Foundation of China (No. 32370568) provided financial support for the study. The funders played no role in study design, data collection and analysis, decision to publish, or preparation of the manuscript.

3. Thank you for your guidance regarding the Data Availability Statement. We have compiled the minimal data set underlying the findings reported in our study, including the raw numerical values used for calculating means, standard deviations, and other reported measures, and have uploaded these data as Supporting Information files (S1_Data.xlsx) alongside our revised manuscript. Accordingly, we have revised the Data Availability Statement in the manuscript to clearly reflect this, and the new statement reads: “The minimal data set underlying the results presented in this study is available within the paper and its Supporting Information files. Specifically, the raw data for all statistical analyses and tables reported in the manuscript are provided in S1_Data.xlsx.” We confirm that the submission now contains all raw data required to replicate the results of the study.

**Response to Editor’s Comments:**

Editor’s comment 1: The first confusion arises from the title of the manuscript: “The genetic polymorphism of HLA-DRB1 and HLA-DQB1 in response to the rising rates of atherosclerosis in Northeast China.” however, authors describe in the abstract their results of serotype HLA-DR7-DQ2?

Author’s response: Thank you for pointing out this inconsistency. We agree that the mixing of genotypic (HLA-DRB1/DQB1 alleles) and serotypic (HLA-DR7-DQ2) nomenclature can cause confusion. To address this, we have now standardized the terminology throughout the manuscript. In the abstract, we have replaced “serotype HLA-DR7-DQ2” with the corresponding allele-level description: “the HLA-DRB1*07:01-DQB1*02:02 haplotype.” We have also carefully reviewed the entire manuscript to ensure consistent use of allele nomenclature as per current HLA typing standards.

Editor’s comment 2: In these types of studies is very important to have the ancestry data of those haplotypes in the north-east China, DRB1*07:01, DRB1*09:01, DRB1*04:05 (prevalence/ frequency) in the population of Northwest China, the same applies to HLA-DQB1*03:01, -DQB1*02:02, and -DQB1*03:03 alleles.

Author’s response: We appreciate this important suggestion. To provide population context for our findings, we reviewed the available literature on HLA allele frequencies in Northeast Chinese populations.

Several studies have reported HLA allele frequencies in this region. Zhou et al. analyzed high-resolution HLA data from 169,995 Chinese volunteers, including donors from Northeast China, and reported nationwide frequencies of 8.91% for DRB1*07:01, 14.32% for DRB1*09:01, and 4.71% for DRB1*04:05 [1]. A large-scale study of the Liaoning Han population (n=7,823) identified DRB1*09:01, DRB1*15:01, and DRB1*07:01 as the three most frequent alleles at the DRB1 locus [2]. This distribution pattern is supported by an earlier, smaller study in the same population (n=159), which reported frequencies of 10.7% for DRB1*07:01 and 12.8% for DRB1*09:01, along with a frequency of 1.9% for DRB1*04:05 [3]. Consistent with these findings from Liaoning, a study of 13,670 donors in the neighboring province of Heilongjiang also confirmed DRB1*07:01 and DRB1*09:01 among the most frequent alleles, each exceeding 10% [4]. For HLA-DQB1 alleles, Zhou et al. (2015) also provided nationwide frequency data, reporting frequencies of 21.09% for DQB1*03:01, 7.57% for DQB1*02:02, and 15.70% for DQB1*03:03 [1]. Liu et al. (2005) studied a Harbin population (n=100) and reported DQB1*03:01 frequency of 14.51% and DQB1*03:03 frequency of 3.64% in healthy controls [5]. However, specific regional data for DQB1*02:02 in Northeast China remain limited.

Compared to these background frequencies in healthy Northeast Chinese populations, our case group showed a significantly higher frequency of DRB1*07:01 (25.19% vs. an average of 13.51% in controls, *P* < 0.05), suggesting a true disease association rather than population-specific enrichment.

Based on the above evidence, we have added a paragraph in the Discussion section (lines 246–253) summarizing the reported frequencies of the relevant alleles in healthy Northeast Chinese controls. The revised text reads as follows:

“Consistent with reported allele frequencies in the general Northeast Chinese population, which range from 8.91% to 10.70% for DRB1*07:01 and indicate 7.57% for DQB1*02:02 based on nationwide data, our control group showed expected distributions at 13.51% and 14.86%, respectively. In contrast, the case group exhibited a significantly higher frequency of DRB1*07:01 (25.19% vs. 13.51%, *P* < 0.05) and a modestly elevated frequency of DQB1*02:02 (24.44% vs. 14.86%, *P* > 0.05), supporting disease-related enrichment rather than population-specific artifact.”

**References**

1. Zhou XY, Zhu FM, Li JP, Mao W, Zhang DM, Liu ML, et al. High-Resolution Analyses of Human Leukocyte Antigens Allele and Haplotype Frequencies Based on 169,995 Volunteers from the China Bone Marrow Donor Registry Program. PLoS One. 2015;10(9):e0139485.
2. Shao LN, Zhang ST, Yu WJ, Zhou SH, Duan Y, Pan LZ, Wang N, Liu M. High-resolution HLA-A, -B and -DRB1 allele and haplotype frequencies in 7823 Han marrow donors of Liaoning province, China. HLA. 2017;89(5):293-300.
3. Jiang P, Shan XY, Zhang BW, Zhu CF, Shen J, Deng ZH, et al. Polymorphic Analysis of HLA-DRB1 Gene in Chinese Liaoning Han Population by Sequence Specific Primers-PCR and Sequence Specific Oligonucleotide Probes. Yi Chuan. 1999;21(3):5-8.
4. Li X, Ding J, Wang X, Hou L, Liu Y, Liu J. Polymorphism research of HLA-A, B, DRB1 high resolution alleles and haplotypes in population from Heilongjiang. Chinese Journal of Immunology. 2016;32(1):83-89.
5. Liu W, Li WM, Sun NL. Polymorphism of the second exon of human leukocyte antigen-DQA1, -DQB1 gene and genetic susceptibility to idiopathic dilated cardiomyopathy in people of the Han nationality in northern China. Chin Med J. 2005;118(3):238-241.

Editor’s comment 3: “authors should be careful with the description of HLA-DR/DQ, this is not a haplotype!!, is a serotype, this is stated in results section where authors described the results of NGS, I suggest authors homogenize the nomenclature of HLA alleles (lines 169, 179, 182 and 199).”

Author’s response: We sincerely thank the reviewer for this crucial clarification. You are absolutely correct that HLA-DR/DQ refers to serotypes, not haplotypes, and that our results from NGS should be reported using standardized allele nomenclature. We have carefully revised the manuscript accordingly: At lines 169, 179, 182, and 199 (now lines 169, 180, 183, 206), we have replaced all instances of serotype descriptions with the corresponding allele nomenclature.

We have also reviewed the entire manuscript to ensure consistent and accurate use of HLA terminology throughout, including in tables.

Editor’s comment 4: It was not clear the estimation of statistical significancy (p-value) for association of the HLA-DRB1*07:01-DQB1*02:02 haplotype with disease susceptibility, this values must be corrected using the Bonferroni test, which is necessary in this type of studies, and besides, authors should mention if the values shows some kind of trend toward susceptibility for this haplotype.

Author’s response: Thank you very much for your excellent methodological comment regarding the necessity of multiple testing correction in HLA association studies. We fully agree that Bonferroni correction is essential to control for type I errors when analyzing multiple haplotypes.

Following your suggestion, we have re-analyzed our data accordingly. We applied the Bonferroni correction based on the total number of HLA-DRB1-DQB1 haplotypes tested in our study (n = 4). The raw p-value for each haplotype was multiplied by 4, and corrected p-values no more than 0.05 were considered statistically significant. The revised analysis yielded the following results for the HLA-DRB1*07:01-DQB1*02:02 haplotype:

Raw analysis: OR = 2.357 (95% CI: 1.019-5.452), *P* = 0.041

Bonferroni-corrected analysis: corrected *P* = 0.041 × 4 = 0.164

After correction, the association between the HLA-DRB1*07:01-DQB1*02:02 haplotype and disease susceptibility did not retain statistical significance (corrected *P* = 0.164). Nevertheless, the odds ratio (OR = 2.357) and its 95% confidence interval, which remains entirely above 1 (1.019–5.452), point to a consistent trend toward disease susceptibility by indicating a higher frequency of this haplotype in the AS-susceptible group than in controls. Given the limited sample size, particularly in the control group (n = 74), this trend should be interpreted with caution and warrants further validation in larger, independent cohorts.

We have also updated the Results section to report both uncorrected and corrected p-values, and have added a comment in the Discussion acknowledging this trend and the need for cautious interpretation given the sample size and the conservative nature of the Bonferroni correction.

Editor’s comment 5: There is an error in line 212, the haplotype HLA-DRB1*07:01-DQB1*02:02. should be corrected.

Author’s response: Thank you for catching this typographical error. We have corrected line 212 (now line 217) to properly write the haplotype as HLA-DRB1*07:01-DQB1*02:02. We have also carefully reviewed the entire manuscript to ensure all haplotype designations follow the correct format.

Editor’s comment 6: In discussion section, I suggest deleting the next phrase “in murine models, several variants associated with cardiovascular risk have been identified.” This makes confusion due to the clear differences between the murine MHC and the HLA in humans, both complexes are not comparables (line132).

Author’s response: We agree with the reviewer that comparing murine MHC findings directly to human HLA studies can be misleading, as the two systems are not directly comparable. We have therefore deleted the phrase "in murine models, several variants associated with cardiovascular risk have been identified" from the Discussion section. The discussion now focuses solely on human studies to maintain clarity and scientific accuracy.

**Response to Reviewer #1:**

We would like to express our sincere gratitude to the reviewer for the thorough and constructive comments, which have helped us identify key areas for improvement. We have carefully addressed each point and revised the manuscript accordingly.

Reviewer #1 Comment 1: Why, if the article title frames the genetic polymorphism of HLA-DRB1 and HLA-DQB1, do you describe the results in the abstract with the serotype HLA-DR7-DQ2?

Author’s response: We thank the reviewer for pointing out this inconsistency. As we also noted in response to the editor’s comment #1, we agree that mixing genotypic and serotypic nomenclature can cause confusion. To resolve this, we have standardized the terminology throughout the manuscript. In the abstract, we have replaced “serotype HLA-DR7-DQ2” with the corresponding allele-level description: “the HLA-DRB1*07:01-DQB1*02:02 haplotype”. We have also carefully reviewed the entire manuscript to ensure consistent use of allele nomenclature.

Reviewer #1 Comment 2: Line 50, it would be illustrative to create a map that frames the northwest region of China, where the samples originate.

Author’s response: We thank the reviewer for the suggestion to provide a geographical map. While we agree that geographic context is valuable, we believe that a map may not be necessary in this context for the following reasons, which we hope the reviewer will find acceptable.

First, as per standard academic publishing practice, the addition of a new figure would be subject to journal formatting constraints and, more importantly, potential copyright restrictions related to base map sources, which could complicate the publication process.

Second, and more fundamentally, we believe a map is not necessary for scientific clarity in this context. Our study participants were recruited from the Department of Cardiology, Liaoyuan Municipal Central Hospital, which is located in Jilin Province, China. As a regional referral center, this hospital serves not only the local population of Liaoyuan City but also receives patients from surrounding areas across Jilin Province, as well as from neighboring regions in Liaoning and Heilongjiang provinces. This patient catchment area reflects the broader population of Northeast China, a well-established and unambiguous geographical designation comprising the three provinces of Liaoning, Jilin, and Heilongjiang in both Chinese and international literature.

To avoid any confusion with “Northwest China”, we have now revised the Methods section (line 71) to explicitly state: “This study enrolled 209 participants aged 30-60 years (mean age: 40; 136 males and 73 females, Table 1) from the Department of Cardiology, Liaoyuan Municipal Central Hospital of Jilin Province, China, between October 2023 and August 2024. Geographically, Jilin is part of Northeast China, a region comprising the provinces of Liaoning, Jilin, and Heilongjiang.” This textual clarification precisely frames the geographic origin of our samples without the need for a map.

Reviewer #1 Comment 3: Line 62, you cite an article you authored, comparing the miniature pig MHC (SLA), on chromosome 7, with the human MHC (HLA). I do not consider them comparable. You should reconsider this idea.

Author’s response: We thank the reviewer for this critical and important observation. You are absolutely correct that directly comparing the genomic architecture or disease associations of the miniature pig MHC (SLA) with the human MHC (HLA) can be misleading, given their evolutionary divergence and different chromosomal contexts. Our intention in citing our previous work was merely to acknowledge our research background in the MHC field, not to draw a direct functional or genetic comparison. However, we recognize that the current phrasing could easily be misinterpreted. To avoid any confusion and to maintain scientific rigor, we have deleted the reference to our own work and the accompanying sentence from the introduction. The focus of the introduction now remains strictly on human HLA and its relevance to atherosclerosis.

Reviewer #1 Comment 4: Line 66, HLA-DR/DQ, describes serotypes, not the haplotype that you will describe later in the results, obtained by NGS.

Author’s response: We thank the reviewer for this important clarification. You are absolutely correct that HLA-DR/DQ refers to serotypes rather than haplotypes, and that our results obtained by NGS should be reported using standardized allele-level nomenclature. We have carefully revised the manuscript accordingly. At line 66 (now line 63), we have replaced the serotype description with the corresponding allele-level terminology. We have also reviewed the entire manuscript to ensure consistent and accurate use of HLA nomenclature throughout, including in the Results section where the NGS findings are presented.

Reviewer #1 Comment 5: In the section on study participants, I think that the description of the patient group is correct.

Author’s response: We thank the reviewer for this positive feedback on the study participants section.

Reviewer #1 Comment 6: Line 161, The results fail to mention whether the most frequent alleles found in your study population, DRB1*07:01, DRB1*09:01, and DRB1*04:05, are prevalent or very frequent in the population of Northwest China. Line 178, the same applies to the HLA-DQB1*03:01, -DQB1*02:02, and -DQB1*03:03 alleles.

Author’s response: We appreciate this important suggestion. As outlined in our response to the editor's comment 2, we have now addressed this by adding population context. We have added a paragraph in the Discussion section (lines 246-253) that summarizes the reported frequencies of these alleles in healthy Northeast Chinese populations based on a review of the literature. This addition allows readers to compare our case and control frequencies with the expected background frequencies in the region, strengthening the interpretation of our findings. The relevant references have been included in the revised manuscript for further detail.

Reviewer #1 Comment 7: Lines 163, 169, 179, 182 and 199, homogenize the nomenclature of the described HLA alleles.

Author’s response: Thank you for pinpointing these specific locations. We have carefully revised lines 163, 169, 179, 182, and 199 (now lines 163, 169, 180, 183, and 206) to ensure consistent and accurate use of HLA allele nomenclature, as detailed in our responses to similar comments above.

Reviewer #1 Comment 8: Line 190, the significant p-value for the association of the HLA-DRB1*07:01-DQB1*02:02 haplotype with susceptibility to developing the disease must be corrected using the Bonferroni test, which is necessary in this type of study of HLA and its association with disease. If the p-value loses its significance, explain, at least, the trend toward susceptibility for this haplotype.

Author’s response: We are grateful for this rigorous methodological guidance. As described in our response to the editor's comment 4, we have performed the Bonferroni correction. The results section has been updated to reflect both the raw (*P* = 0.041) and corrected (*P* = 0.164) *P*-values. In the discussion, we have added a comment acknowledging that while the association loses statistical significance after correction, the odds ratio (OR=2.357, 95% CI: 1.019-5.452) indicates a consistent trend toward disease susceptibility. We also emphasize the need for cautious interpretation due to the sample size and the conservative nature of the correction.

Reviewer #1 Comment 9: Line 212, correct the haplotype HLA-DRB1*07:01-DQB1*02:02.

Author’s response: Thank you for correcting this oversight. We have corrected line 212 (now line 217) to properly write the haplotype as HLA-DRB1*07:01-DQB1*02:02.

Reviewer #1 Comment 10: Line 231, in the discussion, makes no sense in trying to link this study: "in murine models, several variants associated with cardiovascular risk have been identified." The mouse MHC is H-2 and is located on chromosome 17. It is not comparable to HLA on chromosome 6. You need to remove these lines.

Author’s response: We fully concur with the reviewer that directly comparing murine MHC (H-2) findings to human HLA studies is not appropriate. As we mentioned in our response to the editor's comment 6, we have deleted the phrase "in murine models, several variants associated with cardiovascular risk have been identified" from the Discussion section. The discussion now focuses solely on human studies to maintain clarity and scientific accuracy.

We believe that the revisions have substantially improved the manuscript and hope that it is now suitable for publication in PLOS ONE. Thank you for your consideration.

Sincerely,

Chenhua
