# Supplementary material for: HLA-DRB1 and HLA-DQB1 genetic polymorphisms and susceptibility to coronary atherosclerosis in a Northeast Chinese Population: A case-control study
Source: PLoS One. 2026 Jul 23;21(7):e0353906. doi: 10.1371/journal.pone.0353906 (PMC13395462; doi:10.1371/journal.pone.0353906)
Supplement: Attachment — Submitted filename: Response_to_Reviewers_auresp_2.docx [file pone.0353906.s005.docx]

Dear Editor,

Thank you for giving us the opportunity to revise our manuscript (Manuscript ID: PONE-D-25-52254). We appreciate the time and effort that you and the reviewers have dedicated to providing valuable feedback on our work. We have carefully addressed all the comments raised by the editor and the reviewers, and the changes are highlighted in the revised manuscript. Below is our point-by-point response to each comment.

**Response to Reviewer’s Comments:**

Reviewer’s comment 1: Designation of the HLA entities used in the study.

I suggest the authors reconsider the use the term phenotypic (allelic) frequencies this I not correct if we consider the data presented in the manuscript and in supporting information, in genetics this statement is an issue that double fold when you are working with two loci together, you cannot speak on haplotypes because that requires to identify the chromosome where both alleles, one from DRB1, the other form DQB1, occurs maybe the authors can use the term “phenotypic haplotype” followed by the proper explanation, but “haplotype” is not genetically correct, include phenotype (or genotype) to clarify that the values presented are not allelic frequencies.

Author’s response: We agree with the reviewer that our original use of “haplotype” and “allelic frequencies” was genetically inaccurate. Accordingly, we have replaced “haplotype” with “phenotypic haplotype” throughout the manuscript and removed “allelic frequencies”. We have also added the following explanatory sentence in the Methods section (line 110): “In the absence of family data or molecular phasing, the chromosomal phase of DRB1 and DQB1 allele pairs could not be definitively established. Accordingly, we employ the term “phenotypic haplotype” throughout this study to refer to the co-occurrence of a specific DRB1 allele and a specific DQB1 allele at the individual level, recognizing that these combinations do not necessarily represent true haplotypes in phase. The frequency of each phenotypic haplotype was calculated separately for each group as the number of individuals carrying both the DRB1 and DQB1 alleles of interest, divided by the total number of individuals in each group (n = 135 for the AS-susceptible group; n = 74 for the control group). These phenotypic haplotypes were used for all subsequent association analyses.). These phenotypic haplotypes were used for all subsequent association analyses.”. We believe the manuscript is now scientifically congruent with respect to HLA terminology. Thank you for your constructive critique.

Reviewer’s Comment 2: The second is related to the statistical methods.

In my previous review I suggest the authors that even if this imply more work, that involves considering the two alleles of each individual, then the estimation of allelic and haplotypic frequencies, can duplicate the n, but imply testing for Hardy-Weinberg from the Controls and probably the p values could not be statistically significant but at least can show a tendency (trend)...

Author’s response: We thank the reviewer for this valuable and constructive methodological suggestion. We fully agree that incorporating both alleles per individual and testing for Hardy-Weinberg equilibrium (HWE) are important steps to ensure genotyping data quality, even when statistical significance is not reached.

To address the reviewer’s recommendation regarding allele‑based analysis, we re‑analyzed our data at the allelic level, treating the two alleles at each locus per individual as separate observations. This shifted the unit of analysis from *N* individuals to *2N* alleles. To assess genotyping data quality, we tested Hardy-Weinberg equilibrium (HWE) for HLA-DRB1 and HLA-DQB1 in the control group using the “Basic statistics” tool, available on the HLA-net website (<https://hla-net.eu/tools/basic-statistics/>). The results showed no deviation from HWE for either locus (*P* = 1.0000 for both), indicating that the genotypic distributions were consistent with Mendelian expectations and supporting the reliability of the subsequent allele frequency comparisons.

For each HLA allele, we compared allele frequencies between cases and controls using Fisher's exact test or chi‑square test, as appropriate. The results are presented in Table 2. The lowest *P* value was observed for DRB1*07:01 (*P* = 0.077, corrected *P* = 1.000; OR = 1.994, 95% CI: 0.955-4.164), and the smallest was observed for DQB1*02:02 (*P* = 0.150, corrected *P* = 1.000; OR = 1.739, 95% CI: 0.850-3.558). Neither reached statistical significance, though both trended upward in the AS-susceptible group (DRB1*07:01: 12.59% vs. 6.76%; DQB1*02:02: 12.22% vs. 7.43%). As shown in Table 3, the DRB1*07:01-DQB1*02:02 phenotypic haplotype was more frequent in the AS-susceptible group than in the control group (22.22% vs. 10.81%), with an OR of 2.357 (95% CI: 1.019–5.452). Although the association did not survive Bonferroni correction (corrected *P* = 1.000), the effect size suggests that the signal is unlikely to be a trivial statistical artifact.

We have incorporated these results into the revised manuscript in several sections. In the Methods section, we have added a detailed description of the allele‑frequency comparisons, HWE testing procedures, and haplotype estimation methods. We have also incorporated relevant discussion of these points into the Discussion section. In addition, we have included a Supporting Information file presenting the HWE P-values for the control group.

We believe that the revisions have substantially improved the manuscript and hope that it is now suitable for publication in PLOS ONE. Thank you for your consideration.

Sincerely,

Chenhua
