# Supplementary material for: HLA-DRB1 and HLA-DQB1 genetic polymorphisms and susceptibility to coronary atherosclerosis in a Northeast Chinese Population: A case-control study
Source: PLoS One. 2026 Jul 23;21(7):e0353906. doi: 10.1371/journal.pone.0353906 (PMC13395462; doi:10.1371/journal.pone.0353906)
Supplement: Peer review history

# Peer review: Decision Letter 0

**DOI:** 10.1371/journal.pone.0353906.r001

**Authors:** Cecilia Ximenez

24 Feb 2026

PONE-D-25-52254

HLA-DRB1 and HLA-DQB1 genetic polymorphisms and susceptibility to atherosclerosis in a Northeast Chinese Population: a case-control study

PLOS One

Dear Dr. Chen,

Thank you for submitting your manuscript to PLOS ONE. After careful consideration, we feel that it has merit but does not fully meet PLOS ONE’s publication criteria as it currently stands. Therefore, we invite you to submit a revised version of the manuscript that addresses the points raised during the review process.

Please submit your revised manuscript by Apr 06 2026 11:59PM. If you will need more time than this to complete your revisions, please reply to this message or contact the journal office at plosone@plos.org. When you're ready to submit your revision, log on to https://www.editorialmanager.com/pone/ and select the 'Submissions Needing Revision' folder to locate your manuscript file.

Please include the following items when submitting your revised manuscript:

- A letter that responds to each point raised by the academic editor and reviewer(s). You should upload this letter as a separate file labeled 'Response to Reviewers'.
- A marked-up copy of your manuscript that highlights changes made to the original version. You should upload this as a separate file labeled 'Revised Manuscript with Track Changes'.
- An unmarked version of your revised paper without tracked changes. You should upload this as a separate file labeled 'Manuscript'.

If you would like to make changes to your financial disclosure, please include your updated statement in your cover letter. Guidelines for resubmitting your figure files are available below the reviewer comments at the end of this letter.

If applicable, we recommend that you deposit your laboratory protocols in protocols.io to enhance the reproducibility of your results. Protocols.io assigns your protocol its own identifier (DOI) so that it can be cited independently in the future. For instructions see: https://journals.plos.org/plosone/s/submission-guidelines#loc-laboratory-protocols. Additionally, PLOS ONE offers an option for publishing peer-reviewed Lab Protocol articles, which describe protocols hosted on protocols.io. Read more information on sharing protocols at https://plos.org/protocols?utm_medium=editorial-email&utm_source=authorletters&utm_campaign=protocols.

We look forward to receiving your revised manuscript.

Kind regards,

Cecilia Ximenez, Ph.D. MD

Academic Editor

PLOS One

Journal Requirements:

When submitting your revision, we need you to address these additional requirements.

1. Please ensure that your manuscript meets PLOS ONE's style requirements, including those for file naming. The PLOS ONE style templates can be found at

https://journals.plos.org/plosone/s/file?id=wjVg/PLOSOne_formatting_sample_main_body.pdf and

https://journals.plos.org/plosone/s/file?id=ba62/PLOSOne_formatting_sample_title_authors_affiliations.pdf.

2. Thank you for stating the following financial disclosure:

“National Natural Science Foundation of China, No. 32370568.”

Please state what role the funders took in the study.  If the funders had no role, please state: "The funders had no role in study design, data collection and analysis, decision to publish, or preparation of the manuscript."

If this statement is not correct you must amend it as needed.

Please include this amended Role of Funder statement in your cover letter; we will change the online submission form on your behalf.

3. We note that your Data Availability Statement is currently as follows: [All relevant data are within the manuscript and its Supporting Information files.]

Please confirm at this time whether or not your submission contains all raw data required to replicate the results of your study. Authors must share the “minimal data set” for their submission. PLOS defines the minimal data set to consist of the data required to replicate all study findings reported in the article, as well as related metadata and methods (https://journals.plos.org/plosone/s/data-availability#loc-minimal-data-set-definition).

For example, authors should submit the following data:

- The values behind the means, standard deviations and other measures reported;

- The values used to build graphs;

- The points extracted from images for analysis.

Authors do not need to submit their entire data set if only a portion of the data was used in the reported study.

If your submission does not contain these data, please either upload them as Supporting Information files or deposit them to a stable, public repository and provide us with the relevant URLs, DOIs, or accession numbers. For a list of recommended repositories, please see https://journals.plos.org/plosone/s/recommended-repositories.

4. If the reviewer comments include a recommendation to cite specific previously published works, please review and evaluate these publications to determine whether they are relevant and should be cited. There is no requirement to cite these works unless the editor has indicated otherwise.

Additional Editor Comments (if provided):

The manuscript deals with an interesting subject in the study of “HLA-DRB1 and HLA-DQB1 genetic polymorphisms and susceptibility to atherosclerosis in a Northeast Chinese Population: a case-control study”

The first confusion arises from the title of the manuscript: “The genetic polymorphism of HLA-DRB1 and HLA-DQB1 in response to the rising rates of atherosclerosis in Northeast China.”, however, authors describe in the abstract their results of serotype HLA-DR7-DQ2?

In these types of studies is very important to have the ancestry data of those haplotypes in the north-east China, DRB1*07:01, DRB1*09:01, DRB1*04:05 (prevalence/ frequency) in the population of Northwest China, the same applies to HLA-DQB1*03:01, -DQB1*02:02, and -DQB1*03:03 alleles.

Besides, authors should be careful with the description of HLA-DR/DQ, this is not a haplotype!!, is a serotype, this is stated in results section where authors described the results of NGS, I suggest authors homogenize the nomenclature of HLA alleles (lines 169, 179, 182 and 199). It was not clear the estimation of statistical significancy (p-value) for association of the HLA-DRB1*07:01-DQB1*02:02 haplotype with disease susceptibility, this values must be corrected using the Bonferroni test, which is necessary in this type of studies, and besides, authors should mention if the values shows some kind of trend toward susceptibility for this haplotype.

There is an error in line 212, the haplotype HLA-DRB1*07:01-DQB1*02:02. should be corrected.

In discussion section, I suggest deleting the next phrase "in murine models, several variants associated with cardiovascular risk have been identified." This makes confusion due to the clear differences between the murine MHC and the HLA in humans, both complexes are not comparables (line132)

[Note: HTML markup is below. Please do not edit.]

Reviewers' comments:

Reviewer's Responses to Questions

**Comments to the Author**

1. Is the manuscript technically sound, and do the data support the conclusions?

The manuscript must describe a technically sound piece of scientific research with data that supports the conclusions. Experiments must have been conducted rigorously, with appropriate controls, replication, and sample sizes. The conclusions must be drawn appropriately based on the data presented.

Reviewer #1: Partly

**********

2. Has the statistical analysis been performed appropriately and rigorously?

Reviewer #1: No

**********

3. Have the authors made all data underlying the findings in their manuscript fully available?

The PLOS Data policy requires authors to make all data underlying the findings described in their manuscript fully available without restriction, with rare exception (please refer to the Data Availability Statement in the manuscript PDF file). The data should be provided as part of the manuscript or its supporting information, or deposited to a public repository. For example, in addition to summary statistics, the data points behind means, medians and variance measures should be available. If there are restrictions on publicly sharing data—e.g. participant privacy or use of data from a third party—those must be specified.

Reviewer #1: Yes

**********

4. Is the manuscript presented in an intelligible fashion and written in standard English?

PLOS ONE does not copyedit accepted manuscripts, so the language in submitted articles must be clear, correct, and unambiguous. Any typographical or grammatical errors should be corrected at revision, so please note any specific errors here.

Reviewer #1: Yes

**********

5. Review Comments to the Author

Please use the space provided to explain your answers to the questions above. You may also include additional comments for the author, including concerns about dual publication, research ethics, or publication ethics. (Please upload your review as an attachment if it exceeds 20,000 characters)

Reviewer #1: Why, if the article title frames the genetic polymorphism of HLA-DRB1 and HLA-DQB1, do you describe the results in the abstract with the serotype HLA-DR7-DQ2?

Line 50, it would be illustrative to create a map that frames the northwest region of China, where the samples originate.

Line 62, you cite an article you authored, comparing the miniature pig MHC (SLA), on chromosome 7, with the human MHC (HLA). I do not consider them comparable. You should reconsider this idea.

Line 66, HLA-DR/DQ, describes serotypes, not the haplotype that you will describe later in the results, obtained by NGS.

In the section on study participants, I think that the description of the patient group is correct.

Line 161, The results fail to mention whether the most frequent alleles found in your study population, DRB1*07:01, DRB1*09:01, and DRB1*04:05, are prevalent or very frequent in the population of Northwest China. Line 178, the same applies to the HLA-DQB1*03:01, -DQB1*02:02, and -DQB1*03:03 alleles.

Lines 163, 169, 179, 182 and 199, homogenize the nomenclature of the described HLA alleles.

Line 190, the significant p-value for the association of the HLA-DRB1*07:01-DQB1*02:02 haplotype with susceptibility to developing the disease must be corrected using the Bonferroni test, which is necessary in this type of study of HLA and its association with disease. If the p-value loses its significance, explain, at least, the trend toward susceptibility for this haplotype.

Line 212, correct the haplotype HLA-DRB1*07:01-DQB1*02:02.

Line 231, in the discussion, makes no sense in trying to link this study: "in murine models, several variants associated with cardiovascular risk have been identified." The mouse MHC is H-2 and is located on chromosome 17. It is not comparable to HLA on chromosome 6. You need to remove these lines.

**********

6. PLOS authors have the option to publish the peer review history of their article (what does this mean?). If published, this will include your full peer review and any attached files.

If you choose “no”, your identity will remain anonymous but your review may still be made public.

**Do you want your identity to be public for this peer review?**  For information about this choice, including consent withdrawal, please see our Privacy Policy.

Reviewer #1: **Yes:**  Eric G. Hernandez

**********

[NOTE: If reviewer comments were submitted as an attachment file, they will be attached to this email and accessible via the submission site. Please log into your account, locate the manuscript record, and check for the action link "View Attachments". If this link does not appear, there are no attachment files.]

To ensure your figures meet our technical requirements, please review our figure guidelines: https://journals.plos.org/plosone/s/figures

You may also use PLOS’s free figure tool, NAAS, to help you prepare publication quality figures: https://journals.plos.org/plosone/s/figures#loc-tools-for-figure-preparation.

NAAS will assess whether your figures meet our technical requirements by comparing each figure against our figure specifications.

# Author comment: Author response to Decision Letter 1

**DOI:** 10.1371/journal.pone.0353906.r002

6 Apr 2026

Dear Editor,

Thank you for giving us the opportunity to revise our manuscript (Manuscript ID: PONE-D-25-52254). We appreciate the time and effort that you and the reviewers have dedicated to providing valuable feedback on our work. We have carefully addressed all the comments raised by the editor and the reviewers, and the changes are highlighted in the revised manuscript. Below is our point-by-point response to each comment.

Response to Journal Requirements�

1. We have thoroughly reviewed our submission against the PLOS ONE style templates and guidelines. We confirm that the manuscript and all associated files now meet the journal’s formatting and file naming requirements.

2. Role of Funder Statement:

The National Natural Science Foundation of China (No. 32370568) provided financial support for the study. The funders played no role in study design, data collection and analysis, decision to publish, or preparation of the manuscript.

3. Thank you for your guidance regarding the Data Availability Statement. We have compiled the minimal data set underlying the findings reported in our study, including the raw numerical values used for calculating means, standard deviations, and other reported measures, and have uploaded these data as Supporting Information files (S1_Data.xlsx) alongside our revised manuscript. Accordingly, we have revised the Data Availability Statement in the manuscript to clearly reflect this, and the new statement reads: “The minimal data set underlying the results presented in this study is available within the paper and its Supporting Information files. Specifically, the raw data for all statistical analyses and tables reported in the manuscript are provided in S1_Data.xlsx.” We confirm that the submission now contains all raw data required to replicate the results of the study.

Response to Editor’s Comments:

Editor’s comment 1: The first confusion arises from the title of the manuscript: “The genetic polymorphism of HLA-DRB1 and HLA-DQB1 in response to the rising rates of atherosclerosis in Northeast China.” however, authors describe in the abstract their results of serotype HLA-DR7-DQ2?

Author’s response: Thank you for pointing out this inconsistency. We agree that the mixing of genotypic (HLA-DRB1/DQB1 alleles) and serotypic (HLA-DR7-DQ2) nomenclature can cause confusion. To address this, we have now standardized the terminology throughout the manuscript. In the abstract, we have replaced “serotype HLA-DR7-DQ2” with the corresponding allele-level description: “the HLA-DRB1*07:01-DQB1*02:02 haplotype.” We have also carefully reviewed the entire manuscript to ensure consistent use of allele nomenclature as per current HLA typing standards.

Editor’s comment 2: In these types of studies is very important to have the ancestry data of those haplotypes in the north-east China, DRB1*07:01, DRB1*09:01, DRB1*04:05 (prevalence/ frequency) in the population of Northwest China, the same applies to HLA-DQB1*03:01, -DQB1*02:02, and -DQB1*03:03 alleles.

Author’s response: We appreciate this important suggestion. To provide population context for our findings, we reviewed the available literature on HLA allele frequencies in Northeast Chinese populations.

Several studies have reported HLA allele frequencies in this region. Zhou et al. analyzed high-resolution HLA data from 169,995 Chinese volunteers, including donors from Northeast China, and reported nationwide frequencies of 8.91% for DRB1*07:01, 14.32% for DRB1*09:01, and 4.71% for DRB1*04:05 [1]. A large-scale study of the Liaoning Han population (n=7,823) identified DRB1*09:01, DRB1*15:01, and DRB1*07:01 as the three most frequent alleles at the DRB1 locus [2]. This distribution pattern is supported by an earlier, smaller study in the same population (n=159), which reported frequencies of 10.7% for DRB1*07:01 and 12.8% for DRB1*09:01, along with a frequency of 1.9% for DRB1*04:05 [3]. Consistent with these findings from Liaoning, a study of 13,670 donors in the neighboring province of Heilongjiang also confirmed DRB1*07:01 and DRB1*09:01 among the most frequent alleles, each exceeding 10% [4]. For HLA-DQB1 alleles, Zhou et al. (2015) also provided nationwide frequency data, reporting frequencies of 21.09% for DQB1*03:01, 7.57% for DQB1*02:02, and 15.70% for DQB1*03:03 [1]. Liu et al. (2005) studied a Harbin population (n=100) and reported DQB1*03:01 frequency of 14.51% and DQB1*03:03 frequency of 3.64% in healthy controls [5]. However, specific regional data for DQB1*02:02 in Northeast China remain limited.

Compared to these background frequencies in healthy Northeast Chinese populations, our case group showed a significantly higher frequency of DRB1*07:01 (25.19% vs. an average of 13.51% in controls, P < 0.05), suggesting a true disease association rather than population-specific enrichment.

Based on the above evidence, we have added a paragraph in the Discussion section (lines 246–253) summarizing the reported frequencies of the relevant alleles in healthy Northeast Chinese controls. The revised text reads as follows:

“Consistent with reported allele frequencies in the general Northeast Chinese population, which range from 8.91% to 10.70% for DRB1*07:01 and indicate 7.57% for DQB1*02:02 based on nationwide data, our control group showed expected distributions at 13.51% and 14.86%, respectively. In contrast, the case group exhibited a significantly higher frequency of DRB1*07:01 (25.19% vs. 13.51%, P < 0.05) and a modestly elevated frequency of DQB1*02:02 (24.44% vs. 14.86%, P > 0.05), supporting disease-related enrichment rather than population-specific artifact.”

References

1. Zhou XY, Zhu FM, Li JP, Mao W, Zhang DM, Liu ML, et al. High-Resolution Analyses of Human Leukocyte Antigens Allele and Haplotype Frequencies Based on 169,995 Volunteers from the China Bone Marrow Donor Registry Program. PLoS One. 2015;10(9):e0139485.

2. Shao LN, Zhang ST, Yu WJ, Zhou SH, Duan Y, Pan LZ, Wang N, Liu M. High-resolution HLA-A, -B and -DRB1 allele and haplotype frequencies in 7823 Han marrow donors of Liaoning province, China. HLA. 2017;89(5):293-300.

3. Jiang P, Shan XY, Zhang BW, Zhu CF, Shen J, Deng ZH, et al. Polymorphic Analysis of HLA-DRB1 Gene in Chinese Liaoning Han Population by Sequence Specific Primers-PCR and Sequence Specific Oligonucleotide Probes. Yi Chuan. 1999;21(3):5-8.

4. Li X, Ding J, Wang X, Hou L, Liu Y, Liu J. Polymorphism research of HLA-A, B, DRB1 high resolution alleles and haplotypes in population from Heilongjiang. Chinese Journal of Immunology. 2016;32(1):83-89.

5. Liu W, Li WM, Sun NL. Polymorphism of the second exon of human leukocyte antigen-DQA1, -DQB1 gene and genetic susceptibility to idiopathic dilated cardiomyopathy in people of the Han nationality in northern China. Chin Med J. 2005;118(3):238-241.

Editor’s comment 3: “authors should be careful with the description of HLA-DR/DQ, this is not a haplotype!!, is a serotype, this is stated in results section where authors described the results of NGS, I suggest authors homogenize the nomenclature of HLA alleles (lines 169, 179, 182 and 199).”

Author’s response: We sincerely thank the reviewer for this crucial clarification. You are absolutely correct that HLA-DR/DQ refers to serotypes, not haplotypes, and that our results from NGS should be reported using standardized allele nomenclature. We have carefully revised the manuscript accordingly: At lines 169, 179, 182, and 199 (now lines 169, 180, 183, 206), we have replaced all instances of serotype descriptions with the corresponding allele nomenclature.

We have also reviewed the entire manuscript to ensure consistent and accurate use of HLA terminology throughout, including in tables.

Editor’s comment 4: It was not clear the estimation of statistical significancy (p-value) for association of the HLA-DRB1*07:01-DQB1*02:02 haplotype with disease susceptibility, this values must be corrected using the Bonferroni test, which is necessary in this type of studies, and besides, authors should mention if the values shows some kind of trend toward susceptibility for this haplotype.

Author’s response: Thank you very much for your excellent methodological comment regarding the necessity of multiple testing correction in HLA association studies. We fully agree that Bonferroni correction is essential to control for type I errors when analyzing multiple haplotypes.

Following your suggestion, we have re-analyzed our data accordingly. We applied the Bonferroni correction based on the total number of HLA-DRB1-DQB1 haplotypes tested in our study (n = 4). The raw p-value for each haplotype was multiplied by 4, and corrected p-values no more than 0.05 were considered statistically significant. The revised analysis yielded the following results for the HLA-DRB1*07:01-DQB1*02:02 haplotype:

Raw analysis: OR = 2.357 (95% CI: 1.019-5.452), P = 0.041

Bonferroni-corrected analysis: corrected P = 0.041 × 4 = 0.164

After correction, the association between the HLA-DRB1*07:01-DQB1*02:02 haplotype and disease susceptibility did not retain statistical significance (corrected P = 0.164). Nevertheless, the odds ratio (OR = 2.357) and its 95% confidence interval, which remains entirely above 1 (1.019–5.452), point to a consistent trend toward disease susceptibility by indicating a higher frequency of this haplotype in the AS-susceptible group than in controls. Given the limited sample size, particularly in the control group (n = 74), this trend should be interpreted with caution and warrants further validation in larger, independent cohorts.

We have also updated the Results section to report both uncorrected and corrected p-values, and have added a comment in the Discussion acknowledging this trend and the need for cautious interpretation given the sample size and the conservative nature of the Bonferroni correction.

Editor’s comment 5: There is an error in line 212, the haplotype HLA-DRB1*07:01-DQB1*02:02. should be corrected.

Author’s response: Thank you for catching this typographical error. We have corrected line 212 (now line 217) to properly write the haplotype as HLA-DRB1*07:01-DQB1*02:02. We have also carefully reviewed the entire manuscript to ensure all haplotype designations follow the correct format.

Editor’s comment 6: In discussion section, I suggest deleting the next phrase “in murine models, several variants associated with cardiovascular risk have been identified.” This makes confusion due to the clear differences between the murine MHC and the HLA in humans, both complexes are not comparables (line132).

Author’s response: We agree with the reviewer that comparing murine MHC findings directly to human HLA studies can be misleading, as the two systems are not directly comparable. We have therefore deleted the phrase "in murine models, several variants associated with cardiovascular risk have been identified" from the Discussion section. The discussion now focuses solely on human studies to maintain clarity and scientific accuracy.

Response to Reviewer #1:

We would like to express our sincere gratitude to the reviewer for the thorough and constructive comments, which have helped us identify key areas for improvement. We have carefully addressed each point and revised the manuscript accordingly.

Reviewer #1 Comment 1: Why, if the article title frames the genetic polymorphism of HLA-DRB1 and HLA-DQB1, do you describe the results in the abstract with the serotype HLA-DR7-DQ2?

Author’s response: We thank the reviewer for pointing out this inconsistency. As we also noted in response to the editor’s comment #1, we agree that mixing genotypic and serotypic nomenclature can cause confusion. To resolve this, we have standardized the terminology throughout the manuscript. In the abstract, we have replaced “serotype HLA-DR7-DQ2” with the corresponding allele-level description: “the HLA-DRB1*07:01-DQB1*02:02 haplotype”. We have also carefully reviewed the entire manuscript to ensure consistent use of allele nomenclature.

Reviewer #1 Comment 2: Line 50, it would be illustrative to create a map that frames the northwest region of China, where the samples originate.

Author’s response: We thank the reviewer for the suggestion to provide a geographical map. While we agree that geographic context is valuable, we believe that a map may not be necessary in this context for the following reasons, which we hope the reviewer will find acceptable.

First, as per standard academic publishing practice, the addition of a new figure would be subject to journal formatting constraints and, more importantly, potential copyright restrictions related to base map sources, which could complicate the publication process.

Second, and more fundamentally, we believe a map is not necessary for scientific clarity in this context. Our study participants were recruited from the Department of Cardiology, Liaoyuan Municipal Central Hospital, which is located in Jilin Province, China. As a regional referral center, this hospital serves not only the local population of Liaoyuan City but also receives patients from surrounding areas across Jilin Province, as well as from neighboring regions in Liaoning and Heilongjiang provinces. This patient catchment area reflects the broader population of Northeast China, a well-established and unambiguous geographical designation comprising the three provinces of Liaoning, Jilin, and Heilongjiang in both Chinese and international literature.

To avoid any confusion with “Northwest China”, we have now revised the Methods section (line 71) to explicitly state: “This study enrolled 209 participants aged 30-60 years (mean age: 40; 136 males and 73 females, Table 1) from the Department of Cardiology, Liaoyuan Municipal Central Hospital of Jilin Province, China, between October 2023 and August 2024. Geographically, Jilin is part of Northeast China, a region comprising the provinces of Liaoning, Jilin, and Heilongjiang.” This textual clarification precisely frames the geographic origin of our samples without the need for a map.

Reviewer #1 Comment 3: Line 62, you cite an article you authored, comparing the miniature pig MHC (SLA), on chromosome 7, with the human MHC (HLA). I do not consider them comparable. You should reconsider this idea.

Author’s response: We thank the reviewer for this critical and important observation. You are absolutely correct that directly comparing the genomic architecture or disease associations of the miniature pig MHC (SLA) with the human MHC (HLA) can be misleading, given their evolutionary divergence and different chromosomal contexts. Our intention in citing our previous work was merely to acknowledge our research background in the MHC field, not to draw a direct functional or genetic comparison. However, we recognize that the current phrasing could easily be misinterpreted. To avoid any confusion and to maintain scientific rigor, we have deleted the reference to our own work and the accompanying sentence from the introduction. The focus of the introduction now remains strictly on human HLA and its relevance to atherosclerosis.

Reviewer #1 Comment 4: Line 66, HLA-DR/DQ, describes serotypes, not the haplotype that you will describe later in the results, obtained by NGS.

Author’s response: We thank the reviewer for this important clarification. You are absolutely correct that HLA-DR/DQ refers to serotypes rather than haplotypes, and that our results obtained by NGS should be reported using standardized allele-level nomenclature. We have carefully revised the manuscript accordingly. At line 66 (now line 63), we have replaced the serotype description with the corresponding allele-level terminology. We have also reviewed the entire manuscript to ensure consistent and accurate use of HLA nomenclature throughout, including in the Results section where the NGS findings are presented.

Reviewer #1 Comment 5: In the section on study participants, I think that the description of the patient group is correct.

Author’s response: We thank the reviewer for this positive feedback on the study participants section.

Reviewer #1 Comment 6: Line 161, The results fail to mention whether the most frequent alleles found in your study population, DRB1*07:01, DRB1*09:01, and DRB1*04:05, are prevalent or very frequent in the population of

Attachment Submitted filename: Response to Reviewers.docx

# Peer review: Decision Letter 1

**DOI:** 10.1371/journal.pone.0353906.r003

**Authors:** Cecilia Ximenez

1 Jun 2026

PONE-D-25-52254R1

HLA-DRB1 and HLA-DQB1 genetic polymorphisms and susceptibility to coronary atherosclerosis in a Northeast Chinese Population: a case-control study

PLOS One

Dear Dr. Chen,

Thank you for submitting your manuscript to PLOS ONE. After careful consideration, we feel that it has merit but does not fully meet PLOS ONE’s publication criteria as it currently stands. Therefore, we invite you to submit a revised version of the manuscript that addresses the points raised during the review process.

==============================

**ACADEMIC EDITOR:**

I think authors improve the previous version, however they need to read carefully the reviewer’s suggestions , they have options to make their work scientifically congruent.

1. Designation of the HLA entities used in the study.

I suggest   the authors reconsider the use the term  phenotypic (allelic) frequencies this I not correct if we consider the data presented in the manuscript and in supporting information, in genetics this statement is an issue that double fold when you are working with two loci together, you cannot speak on haplotypes because that requires to identify the chromosome where both alleles, one from DRB1, the other form DQB1, occurs maybe the authors can use the term "phenotypic haplotype"  followed by the proper explanation, but "haplotype" is not genetically  correct, include phenotype (or genotype) to clarify that the values presented are not allelic frequencies.

1. The second is related to the statistical methods.

In my previous review I suggest the authors that even if this imply more work, that involves considering the two alleles of each individual, then the estimation of allelic and haplotypic frequencies, can  duplicate the n, but   imply testing for Hardy-Weinberg from the Controls and probably the p values could not be statistically significant but at least can show a tendency (trend)...

==============================

Please submit your revised manuscript by Jul 16 2026 11:59PM. If you will need more time than this to complete your revisions, please reply to this message or contact the journal office at plosone@plos.org. When you're ready to submit your revision, log on to https://www.editorialmanager.com/pone/ and select the 'Submissions Needing Revision' folder to locate your manuscript file.

Please include the following items when submitting your revised manuscript:

- A letter that responds to each point raised by the academic editor and reviewer(s). You should upload this letter as a separate file labeled 'Response to Reviewers'.
- A marked-up copy of your manuscript that highlights changes made to the original version. You should upload this as a separate file labeled 'Revised Manuscript with Track Changes'.
- An unmarked version of your revised paper without tracked changes. You should upload this as a separate file labeled 'Manuscript'.

If you would like to make changes to your financial disclosure, please include your updated statement in your cover letter. Guidelines for resubmitting your figure files are available below the reviewer comments at the end of this letter.

If applicable, we recommend that you deposit your laboratory protocols in protocols.io to enhance the reproducibility of your results. Protocols.io assigns your protocol its own identifier (DOI) so that it can be cited independently in the future. For instructions see: https://journals.plos.org/plosone/s/submission-guidelines#loc-laboratory-protocols. Additionally, PLOS ONE offers an option for publishing peer-reviewed Lab Protocol articles, which describe protocols hosted on protocols.io. Read more information on sharing protocols at https://plos.org/protocols?utm_medium=editorial-email&utm_source=authorletters&utm_campaign=protocols.

As the corresponding author, your ORCID iD is verified in the submission system and will appear in the published article. PLOS supports the use of ORCID, and we encourage all coauthors to register for an ORCID iD and use it as well. Please encourage your coauthors to verify their ORCID iD within the submission system before final acceptance, as unverified ORCID iDs will not appear in the published article. *Only*  the individual author can complete the verification step; PLOS staff *cannot*  verify ORCID iDs on behalf of authors.

We look forward to receiving your revised manuscript.

Kind regards,

Cecilia Ximenez, Ph.D. MD

Academic Editor

PLOS One

**Journal Requirements:**

1. If the reviewer comments include a recommendation to cite specific previously published works, please review and evaluate these publications to determine whether they are relevant and should be cited. There is no requirement to cite these works unless the editor has indicated otherwise.

2. Please review your reference list to ensure that it is complete and correct. If you have cited papers that have been retracted, please include the rationale for doing so in the manuscript text, or remove these references and replace them with relevant current references. Any changes to the reference list should be mentioned in the rebuttal letter that accompanies your revised manuscript. If you need to cite a retracted article, indicate the article’s retracted status in the References list and also include a citation and full reference for the retraction notice.

**Additional Editor Comments:**

I think authors improve the previous version, however they need to read carefully the reviewer’s suggestions , they have options to make their work scientifically congruent.

1- Designation of the HLA entities used in the study.

I suggest the authors reconsider the use the term phenotypic (allelic) frequencies this I not correct if we consider the data presented in the manuscript and in supporting information, in genetics this statement is an issue that double fold when you are working with two loci together, you cannot speak on haplotypes because that requires to identify the chromosome where both alleles, one from DRB1, the other form DQB1, occurs maybe the authors can use the term "phenotypic haplotype" followed by the proper explanation, but "haplotype" is not genetically correct, include phenotype (or genotype) to clarify that the values presented are not allelic frequencies.

2- The second is related to the statistical methods.

In my previous review I suggest the authors that even if this imply more work, that involves considering the two alleles of each individual, then the estimation of allelic and haplotypic frequencies, can duplicate the n, but imply testing for Hardy-Weinberg from the Controls and probably the p values could not be statistically significant but at least can show a tendency (trend)

[Note: HTML markup is below. Please do not edit.]

Reviewers' comments:

Reviewer's Responses to Questions

**Comments to the Author**

1. If the authors have adequately addressed your comments raised in a previous round of review and you feel that this manuscript is now acceptable for publication, you may indicate that here to bypass the “Comments to the Author” section, enter your conflict of interest statement in the “Confidential to Editor” section, and submit your "Accept" recommendation.

Reviewer #1: All comments have been addressed

Reviewer #2: (No Response)

**********

2. Is the manuscript technically sound, and do the data support the conclusions?

The manuscript must describe a technically sound piece of scientific research with data that supports the conclusions. Experiments must have been conducted rigorously, with appropriate controls, replication, and sample sizes. The conclusions must be drawn appropriately based on the data presented.

Reviewer #1: Yes

Reviewer #2: Partly

**********

3. Has the statistical analysis been performed appropriately and rigorously?

Reviewer #1: Yes

Reviewer #2: No

**********

4. Have the authors made all data underlying the findings in their manuscript fully available?

The PLOS Data policy requires authors to make all data underlying the findings described in their manuscript fully available without restriction, with rare exception (please refer to the Data Availability Statement in the manuscript PDF file). The data should be provided as part of the manuscript or its supporting information, or deposited to a public repository. For example, in addition to summary statistics, the data points behind means, medians and variance measures should be available. If there are restrictions on publicly sharing data—e.g. participant privacy or use of data from a third party—those must be specified.

Reviewer #1: Yes

Reviewer #2: Yes

**********

5. Is the manuscript presented in an intelligible fashion and written in standard English?

PLOS ONE does not copyedit accepted manuscripts, so the language in submitted articles must be clear, correct, and unambiguous. Any typographical or grammatical errors should be corrected at revision, so please note any specific errors here.

Reviewer #1: Yes

Reviewer #2: Yes

**********

6. Review Comments to the Author

Please use the space provided to explain your answers to the questions above. You may also include additional comments for the author, including concerns about dual publication, research ethics, or publication ethics. (Please upload your review as an attachment if it exceeds 20,000 characters)

Reviewer #1: I believe that the authors heeded and added to their manuscript the observations and recommendations given, and I consider it suitable for publication in PLOS ONE.

Reviewer #2: The manuscript presents a case control study of atherosclerosis in a Northeast Chinese Population. I found the manuscript interesting but I do found some issues that need to be addressed before it is suitable for publication. The first one refers to the designation of the HLA entities used in the study. The second is somewhat related but concerns the statistical methods appropriate.

The genes of the HLA are autosomal and exist in two copies in each individual, hence a sample size of 2n genes. When we speak of allele frequencies we refer to those not the phenotypic frequencies (sample size of n). Given the data presented in the manuscript and in supporting information, clearly the authors are using phenotypic (allelic) frequencies. This is not a problem by itself but deserves correction, even though I know many published articles use the inappropriate designation of allele frequencies. This same (formal genetics) issue is double fold with two loci together, we cannot speak oh haplotypes because that requires to identify the actual phase (the chromosome where both alleles, one from DRB1, the other form DQB1, occur). I would argue the most correct designation is "two-locus phenotype", I do not like but could accept "phenotypic haplotype" if an expalnatin of the intended term is provided, but "haplotype" is clearly misleading. In my opinion the authors have to options: 1. correct the designations to include phenotype (or genotype) so that it si clear that the values presented are not allelic frequencies), 2. do a proper (formal) genetic study, that involves considering the two alleles of each individual (that doubles the sample size) then the estimation of allelic and haplotypic frequencies -this also implies testing for Hardy-Weinberg from the Controls. Then use those to complete the comparison. I guess the second option requires much more work from the authors and although it would provide more statistical power (the sample size doubles) it may not be enough to get significant results.

The second issue is with the comparison themselves. The table 2 shows values for 4 two-locus phenotypes, why 4?, why these 4? why not just one? Isn't this sherry-picking? The same question apply to table 4.

Finally a few minor details:

l64: *a* Northeast Chinese population rather than *the*

l90: no n=209 in the table title

l120: precise criteria for when Chi² or Fisher are appropriate (or not)

l187: LD between DRB1 and DQB1, some ref needed here

This would be minor revision unless the authors decide to use the two alleles carried by each individual (and explore their possible homozygosity in association with Angiography status).

**********

7. PLOS authors have the option to publish the peer review history of their article (what does this mean?). If published, this will include your full peer review and any attached files.

If you choose “no”, your identity will remain anonymous but your review may still be made public.

**Do you want your identity to be public for this peer review?**  For information about this choice, including consent withdrawal, please see our Privacy Policy.

Reviewer #1: **Yes:**  Eric G. Hernandez

Reviewer #2: **Yes:**  José Manuel Nunes

**********

[NOTE: If reviewer comments were submitted as an attachment file, they will be attached to this email and accessible via the submission site. Please log into your account, locate the manuscript record, and check for the action link "View Attachments". If this link does not appear, there are no attachment files.]

To ensure your figures meet our technical requirements, please review our figure guidelines: https://journals.plos.org/plosone/s/figures

You may also use PLOS’s free figure tool, NAAS, to help you prepare publication quality figures: https://journals.plos.org/plosone/s/figures#loc-tools-for-figure-preparation.

NAAS will assess whether your figures meet our technical requirements by comparing each figure against our figure specifications.

# Author comment: Author response to Decision Letter 2

**DOI:** 10.1371/journal.pone.0353906.r004

26 Jun 2026

Response to Reviewer’s Comments:

Reviewer’s comment 1: Designation of the HLA entities used in the study.

I suggest the authors reconsider the use the term phenotypic (allelic) frequencies this I not correct if we consider the data presented in the manuscript and in supporting information, in genetics this statement is an issue that double fold when you are working with two loci together, you cannot speak on haplotypes because that requires to identify the chromosome where both alleles, one from DRB1, the other form DQB1, occurs maybe the authors can use the term “phenotypic haplotype” followed by the proper explanation, but “haplotype” is not genetically correct, include phenotype (or genotype) to clarify that the values presented are not allelic frequencies.

Author’s response: We agree with the reviewer that our original use of “haplotype” and “allelic frequencies” was genetically inaccurate. Accordingly, we have replaced “haplotype” with “phenotypic haplotype” throughout the manuscript and removed “allelic frequencies”. We have also added the following explanatory sentence in the Methods section (line 110): “In the absence of family data or molecular phasing, the chromosomal phase of DRB1 and DQB1 allele pairs could not be definitively established. Accordingly, we employ the term “phenotypic haplotype” throughout this study to refer to the co-occurrence of a specific DRB1 allele and a specific DQB1 allele at the individual level, recognizing that these combinations do not necessarily represent true haplotypes in phase. The frequency of each phenotypic haplotype was calculated separately for each group as the number of individuals carrying both the DRB1 and DQB1 alleles of interest, divided by the total number of individuals in each group (n = 135 for the AS-susceptible group; n = 74 for the control group). These phenotypic haplotypes were used for all subsequent association analyses.). These phenotypic haplotypes were used for all subsequent association analyses.”. We believe the manuscript is now scientifically congruent with respect to HLA terminology. Thank you for your constructive critique.

Reviewer’s Comment 2: The second is related to the statistical methods.

In my previous review I suggest the authors that even if this imply more work, that involves considering the two alleles of each individual, then the estimation of allelic and haplotypic frequencies, can duplicate the n, but imply testing for Hardy-Weinberg from the Controls and probably the p values could not be statistically significant but at least can show a tendency (trend)...

Author’s response: We thank the reviewer for this valuable and constructive methodological suggestion. We fully agree that incorporating both alleles per individual and testing for Hardy-Weinberg equilibrium (HWE) are important steps to ensure genotyping data quality, even when statistical significance is not reached.

To address the reviewer’s recommendation regarding allele‑based analysis, we re‑analyzed our data at the allelic level, treating the two alleles at each locus per individual as separate observations. This shifted the unit of analysis from N individuals to 2N alleles. To assess genotyping data quality, we tested Hardy-Weinberg equilibrium (HWE) for HLA-DRB1 and HLA-DQB1 in the control group using the “Basic statistics” tool, available on the HLA-net website (https://hla-net.eu/tools/basic-statistics/). The results showed no deviation from HWE for either locus (P = 1.0000 for both), indicating that the genotypic distributions were consistent with Mendelian expectations and supporting the reliability of the subsequent allele frequency comparisons.

For each HLA allele, we compared allele frequencies between cases and controls using Fisher's exact test or chi‑square test, as appropriate. The results are presented in Table 2. The lowest P value was observed for DRB1*07:01 (P = 0.077, corrected P = 1.000; OR = 1.994, 95% CI: 0.955-4.164), and the smallest was observed for DQB1*02:02 (P = 0.150, corrected P = 1.000; OR = 1.739, 95% CI: 0.850-3.558). Neither reached statistical significance, though both trended upward in the AS-susceptible group (DRB1*07:01: 12.59% vs. 6.76%; DQB1*02:02: 12.22% vs. 7.43%). As shown in Table 3, the DRB1*07:01-DQB1*02:02 phenotypic haplotype was more frequent in the AS-susceptible group than in the control group (22.22% vs. 10.81%), with an OR of 2.357 (95% CI: 1.019–5.452). Although the association did not survive Bonferroni correction (corrected P = 1.000), the effect size suggests that the signal is unlikely to be a trivial statistical artifact.

We have incorporated these results into the revised manuscript in several sections. In the Methods section, we have added a detailed description of the allele‑frequency comparisons, HWE testing procedures, and haplotype estimation methods. We have also incorporated relevant discussion of these points into the Discussion section. In addition, we have included a Supporting Information file presenting the HWE P-values for the control group.

We believe that the revisions have substantially improved the manuscript and hope that it is now suitable for publication in PLOS ONE. Thank you for your consideration.

Attachment Submitted filename: Response_to_Reviewers_auresp_2.docx

# Editor report: Decision Letter 2

**DOI:** 10.1371/journal.pone.0353906.r005

**Authors:** Cecilia Ximenez

1 Jul 2026

HLA-DRB1 and HLA-DQB1 genetic polymorphisms and susceptibility to coronary atherosclerosis in a Northeast Chinese Population: a case-control study

PONE-D-25-52254R2

Dear Dr.Hua Chen

We’re pleased to inform you that your manuscript has been judged scientifically suitable for publication and will be formally accepted for publication once it meets all outstanding technical requirements.

Within one week, you’ll receive an e-mail detailing the required amendments. When these have been addressed, you’ll receive a formal acceptance letter and your manuscript will be scheduled for publication.

An invoice will be generated when your article is formally accepted. Please note, if your institution has a publishing partnership with PLOS and your article meets the relevant criteria, all or part of your publication costs will be covered. Please make sure your user information is up-to-date by logging into Editorial Manager at Editorial Manager® and clicking the ‘Update My Information' link at the top of the page. For questions related to billing, please contact billing support.

If your institution or institutions have a press office, please notify them about your upcoming paper to help maximize its impact. If they’ll be preparing press materials, please inform our press team as soon as possible -- no later than 48 hours after receiving the formal acceptance. Your manuscript will remain under strict press embargo until 2 pm Eastern Time on the date of publication. For more information, please contact onepress@plos.org.

Kind regards,

Cecilia Ximenez, Ph.D. MD

Academic Editor

PLOS One

# Editor report: Acceptance letter

**DOI:** 10.1371/journal.pone.0353906.r006

**Authors:** Cecilia Ximenez

PONE-D-25-52254R2

PLOS One

Dear Dr. Chen,

I'm pleased to inform you that your manuscript has been deemed suitable for publication in PLOS One. Congratulations! Your manuscript is now being handed over to our production team.

At this stage, our production department will prepare your paper for publication. This includes ensuring the following:

* All references, tables, and figures are properly cited

* All relevant supporting information is included in the manuscript submission,

* There are no issues that prevent the paper from being properly typeset

You will receive further instructions from the production team, including instructions on how to review your proof when it is ready. Please keep in mind that we are working through a large volume of accepted articles, so please give us a few days to review your paper and let you know the next and final steps.

Lastly, if your institution or institutions have a press office, please let them know about your upcoming paper now to help maximize its impact. If they'll be preparing press materials, please inform our press team within the next 48 hours. Your manuscript will remain under strict press embargo until 2 pm Eastern Time on the date of publication. For more information, please contact onepress@plos.org.

You will receive an invoice from PLOS for your publication fee after your manuscript has reached the completed accept phase. If you receive an email requesting payment before acceptance or for any other service, this may be a phishing scheme. Learn how to identify phishing emails and protect your accounts at https://explore.plos.org/phishing.

If we can help with anything else, please email us at customercare@plos.org.

Thank you for submitting your work to PLOS ONE and supporting open access.

Kind regards,

PLOS ONE Editorial Office Staff

on behalf of

Dr. Cecilia Ximenez

Academic Editor

PLOS One
